# Oral health–related quality of life in head and neck cancer: a systematic review

**DOI:** 10.3389/froh.2025.1691065

**Published:** 2025-11-19

**Authors:** Nadisha Piyarathne, Serena Sinclair, Khaled Shawki Elsayed, Chelsea Cook, Ekta Gupta, Rasha Abu-Eid

**Affiliations:** 1 Institute of Dentistry, School of Medicine, Medical Sciences and Nutrition, University of Aberdeen, Aberdeen, United Kingdom; 2 Center for Research in Oral Cancer, Faculty of Dental Sciences, University of Peradeniya, Kandy, Sri Lanka; 3 Oral and Maxillofacial Surgery Department, Horus University, New Damietta, Egypt; 4 NHS Education Scotland, NHS Grampian, Aberdeen, United Kingdom; 5 School of Dentistry, School of Health Sciences, College of Medicine and Health, University of Birmingham, Birmingham, United Kingdom

**Keywords:** oral health related quality of life, oral health impact profile, head and neck cancer, oral cancer, oral potentially malignant disorders

## Abstract

**Objectives:**

Head and Neck Cancer (HNC) is a devastating disease with significant mortality and morbidity. Patients suffer from compromised quality of life, due to the impact of the disease and its treatment on oral health and related functions. The aim of this systematic review was to identify the effects of HNC on oral health related quality of life (OHQoL).

**Methods:**

The protocol followed PRISMA-2020 guidelines. Literature search was conducted in electronic databases (PSYC-INFO, EMBASE, OVID-MEDLINE, SCOPUS, and WEB OF SCIENCE) at three time points, yielding 1198 records. Abstracts and full-texts were screened, and 101 eligible articles were identified. The risk of bias assessment was conducted using Joanna Briggs Institute critical appraisal tools. Narrative data synthesis was conducted under broad themes that influenced OHQoL; patient factors, diagnosis and treatment, and post treatment.

**Results:**

Studies were published between 2001 and 2024, a growing interest in OHQoL research was noted over time. 70.3% of the studies used oral health impact profile (OHIP-14) for OHQoL assessment. Among patient factors, low socioeconomic status, being without a partner and underweight were associated with worse scores. OHQoL varied with anatomical location of HNC, treatment modalities and their side effects such as mucositis and xerostomia. Prosthetic rehabilitation positively influenced OHQoL post-treatment.

**Conclusions:**

OHQoL assessment is critical in HNC patients from diagnosis, during treatment and beyond. It is influenced by factors related to sociodemographic, diagnosis, treatment, reconstruction and rehabilitation. The findings of this study can inform and guide clinicians to update supportive care and existing management of HNC and OPMD patients.

## 1 Introduction

Head and neck cancers (HNC) refer to malignancies arising in oral cavity (including mucosal lip), pharynx, larynx, and paranasal sinuses (1). The incidence of HNC is on the rise globally, with geographical variations due to differences in risk factors (2). Management strategies for HNC include surgery, radiotherapy, chemotherapy, immune therapy and combinations of different therapeutic approaches (3).

Quality of Life (QoL) is a concept that describes an individual's holistic well-being. It is influenced by biological factors, social determinants and interactions with the physical environment. Health related quality of life (HRQoL) assesses the impact of disease status or its treatment on physical, psychological and social well-being of individuals. Oral health related quality of life (OHQoL) is a subset of HRQoL, that focuses on oral health and how it affects well-being. Specifically, OHQoL subjectively evaluates patients’ oral health, and their reported comfort with function when eating, sleeping, and engaging in social interactions. It also evaluates patients’ self-esteem; and satisfaction with their oral health (4–6).

Although OHQoL is a subjective construct, researchers have developed questionnaire-based tools to objectively evaluate OHQoL. Two such commonly used tools are the Oral Health Impact Profile (OHIP) and the Oral Impacts on Daily Performances (OIDP) scales. Slade and Spencer developed the original OHIP questionnaire with 49 items and condensed it to a shorter 14-item version (7). The short form of OHIP is considered to be a useful instrument for use in clinical settings with good reliability, validity and precision (8). OHIP-14 covers seven main domains related to OHQoL, namely functional limitations, physical pain, psychological discomfort, physical disability, psychological disability, social disability, and handicap. Each domain is assessed via two questions and equally weighted. Due to the lack of questions related to esthetics in the OHIP tool, the Orofacial Esthetic Scale (OES) was developed (9). Other tools were also developed to help in the assessment of OHQoL including the Chewing Functional Questionnaire (CFQ) (10), and the Oral Mucositis-specific Quality of Life measure (OMQoL) (11). In 2004, Pace-Balzan and colleagues published a pilot study that used the Liverpool Oral Rehabilitation Questionnaire (LORQ) and subsequently developed the current version consisting of 40 items, LORQ version 3 (LORQv3), which captures patient's perception on oral problems and the success of their prosthetic rehabilitation **(**12). The European Organization for Research and Treatment of Cancer (EORTC) has developed several measurement tools to assess the quality of life in cancer patients. Specific modules to assess quality of life in HNC (EORTC QLQ-HN43/35) and quality of life related to oral health (EORTC QLQ—OH15) are available (13).

Patients with HNC are a group whose oral health and related well-being can be particularly affected due to the disease and its treatment. OHQoL has been researched at various stages of HNC, including at the time of diagnosing precursor lesions (oral potentially malignant disorders), cancer detection, treatment with various modalities, reconstruction and rehabilitation. The association of OHQoL with biological factors, treatment and post treatment related factors are not well documented in HNC patients. This could be due to a limited number of primary studies, heterogeneity on outcome measures and a lack of longitudinal data. In order to bridge this critical research gap, the current systematic review aimed to identify primary research that evaluated the OHQoL in HNC patients. The findings will help clinicians to comprehend various factors associated with OHQoL at different stages of HNC, which will enable appropriate modification of treatment strategies and management protocols to deliver optimum care and support for HNC patients.

## 2 Materials and methods

### 2.1 Protocol development

The study protocol was developed according to Preferred Reporting System for Systematic Reviews (PRISMA—2020) guidelines. The review protocol was not registered at the time of the study inception. The review question was defined according to the SPIDER format as follows: Sample of interest—Head and Neck Cancer, Phenomenon of Interest: Oral Health related Quality of Life, Design: any observational or interventional study designs, Evaluation: evaluation of OHQoL using validated tools, and Research type: primary research. The focused research question addressed in this review is “Which patient related factors, factors associated with diagnosis, treatment and post treatment affect OHQoL in patients with HNC?”.

### 2.2 Literature search

A comprehensive literature search was conducted in five electronic databases (PSYC INFO, EMBASE, OVID MEDLINE, SCOPUS, and WEB OF SCIENCE), to identify relevant literature at three time points. Controlled vocabulary search terms for head and neck cancer and oral health-related quality of life were used. Keywords included (“OHIP*”, OHIP, “Oral health impact profile”, “Oral health quality of life”, “OIDP*”, “Oral impact on daily performance” (Combined with OR) (group 1) AND (Cancer, Precancer*, Malig*, Premalig*, Neoplasms, Tumor*) (Combined with OR) (group 2), both groups were combined with AND Boolean. The detailed search strings used for each of the databases are presented in (Supplementary Table S1).

Literature search was conducted at three time points. First literature search was conducted to capture studies published from the inception of the databases up to January 2020. A second search was conducted in 2022 and captured articles from 2020 onwards. Search three was conducted in September 2024, to identify records published from 2023 up to the search date (September 2024). Three different researchers conducted the literature search. To ensure consistency, the original search was repeated at the time of the second and third searches, and comparisons were made to confirm reproducibility.

### 2.3 Screening and study selection

**Figure 1:**
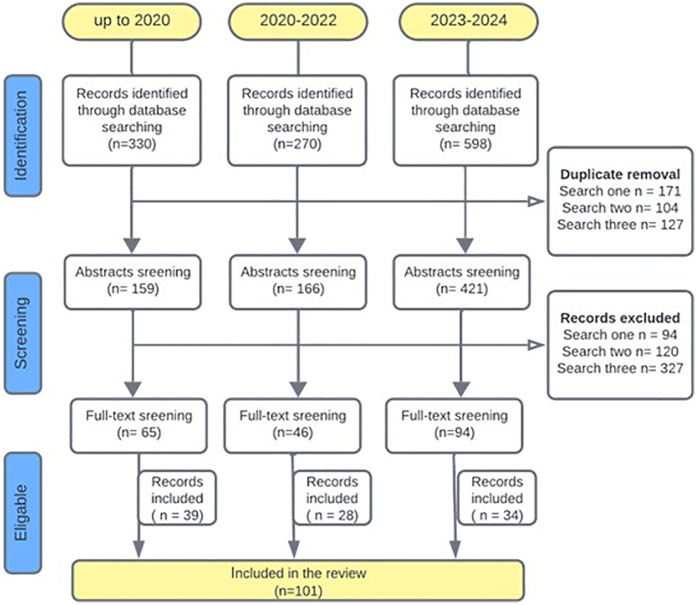
PRISMA flow chart depicting screening and study selection for the systematic review.

All records identified from the literature search were exported and managed through reference management software packages (Endnote and Rayyan.ai). Following duplicate removal, screening was performed at two stages: title and abstract screening and full text screening by independent reviewers using pre-defined selection criteria. The inclusion criteria were (1) studies including patients with HNC, (2) studies with human subjects aged 18 and above, and (3) studies that assessed OHQoL with a standardized tool. The exclusion criteria were (1) full text articles published in languages other than English, (2) studies including non-human participants, (3) studies that did not include HNC patients, (4) studies that did not report OHQoL and (5) case reports, case series, systematic reviews, meta-analysis, and conference proceedings. Details of literature searches and study selection are described in Figure 1, according to a modified PRISMA flow diagram.

### 2.4 Data extraction, quality assessment and data synthesis

The studies which met the inclusion criteria were retrieved and data extraction was performed using a pre-defined bespoke Microsoft Excel spreadsheet by independent reviewers. Any disagreements on screening and data extraction were resolved through discussion and random accuracy checks were performed by RAE and EG. Data extracted from included studies were: first author, manuscript title, year of publication, country where the study was conducted, study design, study groups, diagnosis, sample size, OHQoL assessment tool used, outcomes, any association between studied parameters, conclusions, recommendations, and limitations.

Risk of bias assessment was conducted for the included studies by independent reviewers using Joanna's Briggs Institute (JBI) quality appraisal tools. Since both observational and intervention study designs were included, JBI critical appraisal checklists for analytical cross sectional, case control, cohort and randomized controlled trial designs were used. Scores were presented as the statements marked as ‘Yes’ out of the total number of statements for each tool. Gradings for each study were decided following consensus agreement among the research team. For JBI-cross sectional tool, studies with 8–6 satisfied criteria were rated as good, 5–3 fair and <3 poor, JBI-cohort tool studies with 9–11 satisfied criteria were rated as good, 6–8 fair and <6 poor, for JBI-RCT tool studies satisfying 11–13 criteria were rated as good, 8–10 fair and <8 poor. For JBI-case control tool studies satisfying 10–8 were rated as good, 7–5 fair and less than 5 poor.

The included studies were of heterogenous nature; therefore, a meta-analysis and quantitative synthesis was not considered. A descriptive analysis and thematic data synthesis was conducted under three main headings: (1) patient related factors, (2) diagnosis and treatment, and (3) post treatment.

## 3 Results

### 3.1 Characteristics of the included studies

**Figure 2:**
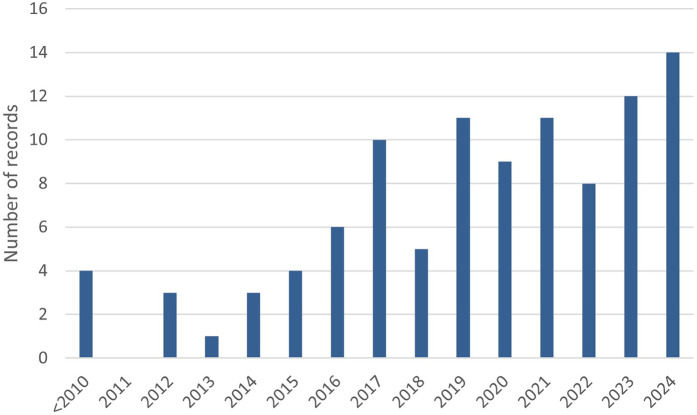
Year-wise distribution of studies included in the systematic review.

A total of *n* = 1,198 records were retrieved from literature searches conducted at three time points as described in Figure 1. Out of these, *n* = 402 duplicates were removed and a total of *n* = 205 full-text articles were screened from which *n* = 101 articles were included. The main reasons for excluding manuscripts following full text screening were: (1) OHQoL was not assessed with many study only assessing health related quality of life, (2) The studies were not relevant to the topic, for example not related to head and neck cancer, (3) Publications that did not provide data such as protocol studies, conference abstracts and reviews. The 101 selected studies were published between 2001 and 2024, and a year-wise breakdown of publications is presented in Figure 2. Data extracted from each included article are presented in (Supplementary Tables S2, S3).

**Figure 3:**
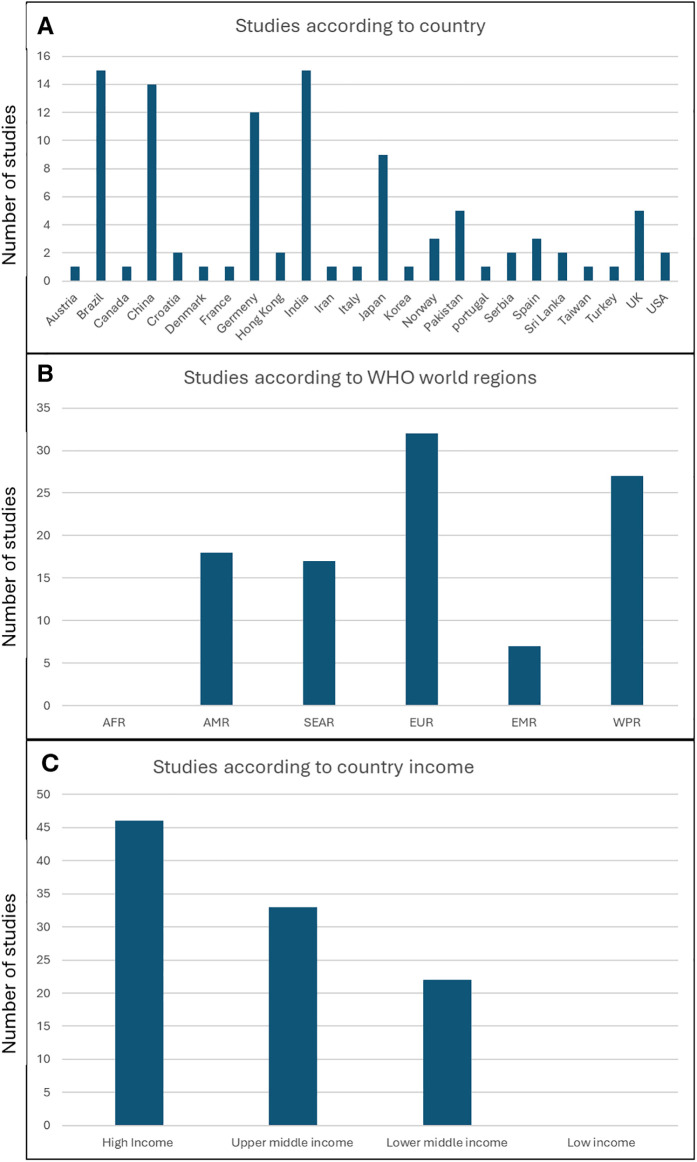
Geographical distribution of the studies **(A)** number of studies per country **(B)** number of studies per wHO world regions **(C)** number of studies according to country income classification. AFR, African region; AMR, region of the Americas; SEAR, South-East Asian region; EUR, European region; EMR, Easter Mediterranean region; WPR, Western Pacific region.

A geographical distribution of the included studies is presented in Figure 3A. A breakdown of countries based on the WHO region classification (Figure 3B) showed that none of the studies were conducted in Africa, and a relatively small number of studies were conducted in South-East Asian region, where the incidence of head and neck cancer is high. Furthermore, based on the World Bank classification of countries, none of the studies were conducted in low-income countries, and the number of studies correlated with the increased income (Figure 2C).

**Figure 4:**
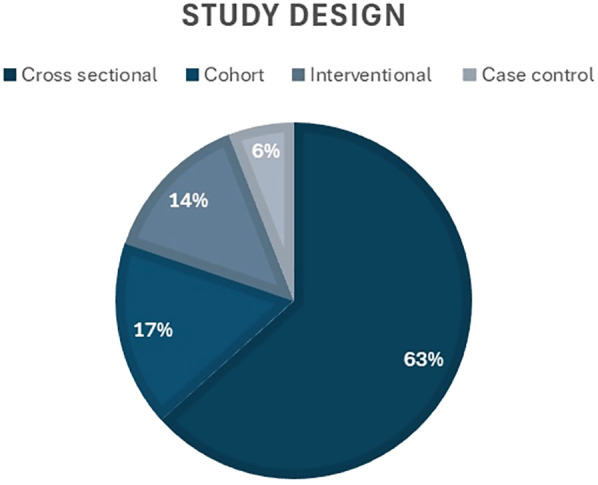
The percentage of included studies classified by study design.

Regarding the study design, the majority of the studies (*n* = 64, 63.4%) were cross-sectional, while 17% (*n* = 17) were cohort, 14% (*n* = 14) used interventional designs, and 6% (*n* = 6) were case control (Figure 4). Thirteen questionnaire tools were used to assess OHQoL, with some studies reporting more than one tool. The frequency and percentage of studies using each tool is presented in Table 1.

**Table 1:** Frequency and percentage of studies that used questionnaire-based tools to assess OHQoL.

Questionnaires used to assess OHQoL	Number of studies	% from total* (*n* = 101)
Oral Health Impact Profile- short version: OHIP-14	71	70.3
Oral Health Impact Profile -long version	16	15.8
Oral Health Impact Profile—edentulous version	3	2.9
Geriatric/Global Oral Health Assessment Index: GOHAI	3	2.9
Liverpool Oral Rehabilitation Questionnaire version 3: LORQv3	6	5.9
Oral Impact on Daily Performance: OIDP	4	3.9
Psychosocial Aspects of Prosthetic Use Scale: PAPUS	1	0.9
Skull Based Inventory: SBI	1	0.9
European Organization for Research and Treatment of Cancer Quality of Life Questionnaire Oral Health Module: EORTC QLQ–OH15	4	3.9
European Organization for Research and Treatment of Cancer Quality of Life Questionnaire Head and Neck Module: EORTC QLQ-H&N	7	6.9
Denture Satisfaction Index: DSI	1	0.9
Xerostomia-related Quality of Life Scale: XQoLS	1	0.9
Oral health values scale	1	0.9

* The percentage is from the total number of studies included. The data in the table do not add up to 100% as some studies have used more than one questionnaire-based tool.

More than two thirds of the studies used oral health impact profile short version (OHIP-14), for OHQoL assessment. This commonly used tool is simple and can be completed in less than 10 min and has been translated and validated in many languages including Tamil, Hindi, Sinhala, Spanish, Serbian, German, Japanese, Chinese, and Urdu demonstrating a good cross-cultural adaptability. The participants rate their problems on a five-point Likert scale coded as never (score 0), hardly ever (1), occasionally (2), fairly often (3) and very often (4). A higher score in OHIP tool indicates poor OHQoL.

The JBI critical appraisal checklists for randomised controlled trials (RCT), cohort, case control and analytical cross-sectional designs were used for quality appraisal. The majority of the studies were graded as good (*n* = 75, 74.3%) with low risk of bias, (*n* = 21, 20.8%) were rated as fair with moderate risk of bias, and (*n* = 5, 5.0%) were rated as poor with high risk of bias. Results of the quality assessment are presented in Supplementary Table S4.

The result summary of OHQoL measures reported in the studies are presented under the following main themes (1) patient related factors, (2) diagnosis and treatment, and (3) post treatment.

### 3.2 Patient-related factors (demographics and risk factors)

In this section, we will summarise the findings from the included studies that assessed OHQoL in relation to patient related factors (demographics) and HNC risk factors.

Regarding the association of OHQoL with sex, contradictory findings were reported. Worse OHQoL was reported in female patients compared to males in some studies (14, 15). On the other hand, a study reported that scores for physical disability, handicap were significantly higher in men than in women following glossectomy (16), and higher functional and physical disability were identified in males compared to females (16, 17). No significant difference was reported in OHQoL according to sex in another study (18).

In relation to age, OHQoL was reported to be worse in older patients compared to younger counterparts, while psychological issues were higher in younger patients (19). On the other hand, younger patients (age <55 years) had a better OHQoL after surgery as compared to older patients (16). Low body mass index (BMI) was a frequent finding in HNC patients (20, 21). Barrios and colleagues reported that malnutrition was a longer-term determinant of worse OHQoL (22). Patients with BMI less than 25 were more likely to have worse overall OHQoL (20).

HNC is a disease common in the low socioeconomic strata (23). Studies have reported associations between OHQoL and patient's social and economic factors (19, 21, 24). In the research by Binnal and colleagues, rural and poor socioeconomic background was associated with worse OHQoL, while urban residents had lower psychological discomfort when compared to rural patients (19). In addition to low socioeconomic status, being widowed and of a non-Caucasian ethnicity were associated with worse OHQoL (25). In support of the observation regarding marital status, married patients reported better OHQoL and mouth opening capacity compared to patients who were not married (21). Low education level, and lower income were identified as factors negatively affecting OHQoL (21, 24).

Risk factors for HNC include smoking, alcohol abuse, smokeless tobacco (SLT), chewing habits (especially betel) and HPV infections (26). Patients who used tobacco reported higher oral health-related social disability than those who never used tobacco (19). A positive association between duration of SLT usage and physical pain was reported, where the pain scores increased with increase duration of SLT use (19). Surprisingly, the same study reported that daily smokers and alcohol drinkers reported better OHQoL scores than occasional users.

### 3.3 Diagnosis and treatment

This section summarises the results related to patient diagnosis, including the stage of the disease and the impact of various treatment modalities on OHQoL.

Out of the included studies, only a small number (*n* = 8, 7.9%) reported data on OHQoL in oral potentially malignant disorders (OPMD) patients. In patients with oral lichen planus (OLP), correlation between OHQoL and disease severity was reported where the OHQoL declined with increasing severity (27–29). In patients with oral epithelial dysplasia, physical pain and psychological disability were the most compromised domains (30). Regarding different treatment modalities for OPMD, laser therapy resulted in significant improvement in OHQoL in leukoplakia patients (31, 32). Intralesional injections of dexamethasone and hyaluronidase produced similar results in oral submucous fibrosis patients (33).

The anatomical location of malignancy influenced OHQoL in HNC patients. Malignancies in the oropharynx and larynx were associated with worse OHQoL compared to lesions in the nasopharynx (24). Similarly, the lowest OHQoL was found in patients with oral cavity tumor site, compared to cancers located in the nasopharynx (34). Among malignancies arising in the oral cavity, tongue and the floor of the mouth reported a poorer OHQoL outcome than malignancies in the buccal mucosa (35). Maxillary defects resulted in better OHQoL outcomes compared to mandibular defects (36). Xiao and colleagues reported that malignancies involving the anterior skull base were more likely to have better OHQoL, than patients who had mid skull base involvement (37).

The primary treatment modality for HNC is surgical resection, followed by primary closure or reconstruction with flaps. Surgery can be combined with radiotherapy and/or chemotherapy. The choice of the treatment/s and their sequence is determined by the clinicians depending on several factors such as extent of tissue involvement of the tumor, co-morbidities and sometimes patient preference. Several studies reported that radiotherapy (RT) as the primary treatment or as an adjuvant to surgical therapy, resulted in a significant deterioration of the OHQoL compared to patients who did not undergo RT (16, 38–45). Patients OHQoL significantly fluctuated with time, during and after radiotherapy (46). Mucositis was a significant contributing factor for worse OHQoL during radiotherapy (39, 47, 48), together with xerostomia (49–51).

Radiation dose and technique impacted OHQoL as well. A 1,000 Gray (Gy) increase in RT dose was associated with a clinically evident change in difficulty of swallowing solid food, dry mouth, sticky saliva, and taste sensation problems (52). Another study reported that when the average dose received by maxillary anterior region is greater than 28.78 Gy, there was a tendency for deterioration in OHQoL (53). Compared to standard three beam RT technique, unilateral and bilateral neck RT with parotid-sparing techniques were successful in preserving salivary output, and lower radiation dose to contralateral parotid glands resulted in fewer xerostomia complaints one year post RT (49). In contrast, no significant variation of OHQoL was found according to the irradiation technique (3D-CRT vs. IMRT) in long-term survivors of HNC (34). Concurrent photobiomodualtion therapy was effective in preventing the negative impacts on OHQoL, mainly in the final stage of RT, with reduction of oral mucositis symptoms (54). In the study by de Oliveira and colleagues, researchers did not find a significant difference in OHQoL in relation to every day and alternate day photobiomodualtion therapy protocols (55). Topical application of pilocarpine spray stimulated whole salivary flow in HNC patients treated with RT (56). Jiang and colleagues introduced an integrated supportive programme for HNC patients undergoing RT, which included face to face education and telephone coaching which resulted in significant improvement in OHQoL and oral hygiene status, compared to standard care (57).

Oral health status of HNC patients influenced OHQoL, where poor oral hygiene resulted in worse OHQoL (21). Caries activity, periodontal disease and incidence of edentulism were high in individuals undergoing RT among HNC patients (58). Patients who suffered from trismus (41, 59), and xerostomia (43, 50) reported worse OHQoL compared to those who did not. Better OHQoL was reported in patients with more posterior functional tooth units (22); however, no association was found between the length of the dental arch and OHQoL post RT in HNC patients (60). Following intensity modulated RT, the compromised salivary flow was persistent with ocular dryness up to 6–24 months (45, 61).

Studies provide evidence regarding association between OHQoL and psychological status. In the study by Hassel and colleagues, compromised OHQoL predisposed to depression and anxiety (62). In a cohort of patients with oral epithelial dysplasia, nearly one fourth of patients were identified with concurrent psychological conditions such as anxiety, depression and emotional distress (30). In addition, the study by Li and colleagues identified that long term HNC survivors had reduced sleep quality. They reported that extensive neck dissection, poor mental health, and psychological disability are associated with poor sleep and suggested that maintaining good OHQoL could promote better sleep in HNC patients (63).

### 3.4 Post treatment

Many studies included in the systematic review assessed OHQoL in HNC patients post treatment. The results are summarised in this section. Reconstruction with flaps and rehabilitation with prosthesis are common clinical sequalae following primary surgical management in HNC patients. Findings from the studies comparing OHQoL outcomes in relation to different reconstruction methods are summarized in Table 2.

**Table 2:** Details of OHQoL outcomes in relation to reconstruction methods in HNC patients.

Study	Surgical location	Reconstruction methods	OHQoL outcomes
Ishida et al. (2015) (90)	Mandible	Osteo-cutaneous flap	Reconstruction to build mandibular continuity produced good results in functionality, compared to reconstruction with soft tissue alone
Brandão et al. (2016) (91)	Mandible	Acrylic resin based surgical guides	Use of surgical guides significantly improved OHQoL in patients.
Li et al. (2016) (92)	Tongue	Pectoralis Major Pedicled Flap (PMPF), and Radial Forearm Free Flap (RFFF)	Worse scores for OHQoL were reported for PMFF compared to RFFF
Wang et al. (2019) (93)	Tongue	Radial Forearm Free Flap (RFFF), Ulnar Forearm Free Flap (UFFF), and Anterolateral Thigh Flap (ALTF)	All reconstruction methods were similar, but ALTF had a slight advantage over the UFFF and RFFF with regards to higher aesthetics and swallowing functions
Yuan et al. (2016) (94)	Tongue	Free Anterolateral Thigh Perforator Flaps (FATPF), and Free Vascularized Forearm Flap (FVFF)	Functional scores improved over 6- 12 months after the surgery, However, there were no significant differences between the two flap types for all domains at 12 months.
Zhu et al. (2017) (95)	Tongue	Radial Forearm Free Flap (RFFF), Anterolateral Thigh Free Flap (ALTFF) and Nasolabial Island Flap (NLIF)	The scores for psychological discomfort in RFFFs and ALTFF flaps were significantly higher than NLIFs. Variable patterns of sensory recovery were observed with the flap type, and NLIF were better in improving OHQoL compared to the other two
Xu et al. (2022) (96)	Tongue	Radial Forearm Free Flap (RFFF), and Groin Soft Tissue Free Flap (GSFF)	The overall score of the OHIP-14 in the GSFF group was lower than that in the RFFF group; however, the difference was not statistically significant. GSFF exhibited a better appearance of the donor site, better mood, psychological status, OHQoL and social relationships. The OHIP-14 scores for psychological discomfort and social disability differed significantly between the RFFF and GSFF groups.
Al-Aroomi et al. (2024) (97)	Oral cavity	Radial Forearm Free Flap (RFFF) and Ulnar Forearm Free Flap (UFFF)	UFFF exhibited a better appearance, social domain, and low decision regret compared with RFFF, indicating that the UFFF may contribute to improving postoperative OHQoL.
Song et al. (2022) (98)	Tongue	Double-Paddle Peroneal Artery Perforator Free Flap (DPAP), and Pedicled Pectoralis Major Myo-cutaneous Flap (PPMMF)	DPAP significantly improved the patients’ OHQOL compared to reconstruction with PPMMF
Zhang et al. (2020) (99)	Tongue	Free Anterolateral Thigh Perforator Flaps (FATP)	Reconstruction with FATP flaps significantly improved the patients’ OHQoL. The best domain scores were for handicap, psychological disability, and social disability. The highest scores were for psychological disability and physical pain.

Several studies reported data on the effects of surgical techniques on OHQoL. A study compared variations of OHQoL according to the type of mandibulectomy, where AT group included defects of mandibular angle and mental tubercle, the Body group included defects involving mandibular body and the MR group included cases involved with marginal resection (64). The study disclosed that the Body group scored significantly higher than the AT group in the OHIP, and a significant difference was noted between the AT group and Body group in the physical and psychological disability domains. However, the body group showed higher stomatognathic performance than the AT group, despite a minor difference in the chewing performance between the MR and the Body groups (64). Not surprisingly, patients with concurrent gastrostomy, and tracheostomy reported worse OHQoL (24).

Several studies reported a significant improvement in all domains of OHQoL with prosthetic rehabilitation following surgery (8, 65–71); where the highest improvement was in psychological disability and handicap domains (65). Coward and colleagues found no statistically significant relations between the surface areas and mean depths of obturators with OHQoL (72). OHQoL improved following prosthetic rehabilitation for both maxillary and mandibular defects; however, the improvement of scores in different OHIP domains were variable between the two groups (67). Regarding the type of prosthesis, overdentures and fixed metal-acrylic resin prostheses were both acceptable treatments, while the implant-retained fixed metal-acrylic resin prostheses was more apt for managing the marginal mandibular defects (73). Implant-supported removable overdentures improved OHQoL outcomes in patients with reconstructed mandibles (74). A study by Stefano and colleagues (2019) provided evidence that palatal augmentation prosthesis is an effective therapeutic remedy to improve OHQoL in patients with absent or reduced lingual mobility following surgery (70). An obturator relined with soft silicone improved masticatory performance and OHQoL post-maxillectomy as opposed to acrylic resin prosthesis (75).

## 4 Discussion

Head and neck cancer is a debilitating disease, affecting vital functions in the body such as mastication, swallowing, phonation, aesthetics and appearance. Therefore, quality of life in affected individuals is a primary concern during disease management and survival. Health and oral health related quality of life is expected to deteriorate in HNC patients compared to disease free individuals; however, determinants of OHQoL in HNC are not well established. Hence, this systematic review aimed to identify factors that impact OHQoL in HNC patients.

Literature search was conducted at three different time points, which captured more than 1,000 records for initial screening. The year-wise distribution of studies indicated that there is a growing interest in OHQoL research. However, studies assessing OHQoL in oral potentially malignant disorders (OPMD) were less than 10%. This highlights a research gap that needs to be addressed. Evidence from OPMD studies indicates a variability of affected OHQoL domains with different clinical OPMD subtypes. Furthermore, OHQoL can be used as a useful adjunct measure to assess treatment response and can be incorporated as a standard clinical endpoint in care pathways for OPMD patients. We identified that OHQoL was used as a measure of the outcome of interventional studies comparing treatment methods for both HNC and OPMD. This is a positive trend, complying with the holistic approach of health as a construct that involves uplifting different domains pertaining to patient well-being, as opposed to absence of disease.

Results of the current review were organized under three main themes; namely OHQoL associated with patient related factors, diagnosis and treatment, and post treatment. Concerning the patient related factors, lower financial and social status were shown to be associated with worse OHQoL. HNC is more common among low socioeconomic groups, and financial strains due to cancer and lack of social support were identified as significant unmet needs in HNC survivors (76, 77). Spousal support was identified as a positive determinant for better OHQoL in HNC patients (21, 25), indicating that caring behavior and emotional support may help to maintain better OHQoL. A study assessing the psychological status of family caregivers of oral cancer patients reported that more than half of the caregivers experienced depression, anxiety, and perceived stress; iterating the need for comprehensive domestic and social support network for patients with HNC (78).

Interestingly, when we looked at the distribution of studies according to the WHO geographical regions and the World Bank classification of countries according to income, we identified a complete absence of studies in the African region and a smaller number of studies in the South-East Asian region, which has a high incidence of head and neck cancer, compared to the European and Western Pacific regions, where more studies on OHQoL in HNC have been conducted. Furthermore, our study showed that there were no studies conducted on this important topic in low-income countries, and that the number of studies correlated with income level in different countries. Given the association between low socioeconomic status and HNC, this clearly identifies a gap that needs to be addressed by conducting more research in the low income countries.

The current review did not identify conclusive evidence on the association of OHQoL with age and sex in HNC patients due to conflicting reports; however, significant evidence was reported for worse OHQoL associated with underweight or low BMI. With the compromised mastication, swallowing and salivary flow, maintaining adequate nutrition and optimum weight is a significant challenge for HNC patients. In agreement with the study reporting association between lower OHQoL and malnourishment in oral cancer patients (22), poor nutrition was a significant determinant of patient dissatisfaction, communication problems and poor decision making in HNC (79). In the study by Widaman and colleagues (2025), researchers reported that HNC patients undergoing professional nutritional assessment with dietician referrals demonstrated better satisfaction with care than those who were without nutritional assessment (79). These findings underscore the importance of incorporating nutritional assessment and dietary counselling in the management protocols for HNC patients.

When it comes to risk factors, we identified an unexpected finding, where daily users of tobacco and alcohol reported better OHQoL scores than occasional users (19). The reasons for these surprising results may be due to the euphoric effects, psychological coping mechanisms and socialization habits of tobacco and alcohol users and should be interpreted with caution.

OHQoL outcomes varied with the anatomical location of the malignancy in HNC. Mid skull base malignancies reported worse OHQoL measures compared to cancers involving anterior skull base (37); poor surgical access and higher number of vital structures in mid skull base may result in more surgical challenges and may compromise the success of the surgical procedure. Following reconstruction, maxillary defects resulted in better OHQoL outcomes compared to mandibular defects (36), this can be due improved blood supply in maxilla, better retention and enhanced acceptance of flaps. Defects of the maxilla, tongue and soft palate in addition to chemoradiotherapy were found to negatively affect the effectiveness of maxillofacial prosthetic treatment, while reconstructive surgery improved OHQoL (80).

Radiotherapy (RT) to the head and neck region resulted in significant deterioration of OHQoL. Radiation induces biological effects such as DNA damage, cell cycle arrest, cell death, and inflammation in irradiated areas and have a negative effect on tissues with rapid turnover. RT in the head and neck region may frequently present with complications such as oral and ocular mucosal irritation (mucositis), bone marrow suppression, fibrosis (in salivary glands leading to reduced salivary secretions), and necrosis (eg: osteoradionecrosis). Reduced salivary flow may cause taste disturbances, increase risk of caries and tooth loss, inflammation and irritation in oral mucosa can result in pain and burning sensation which may predispose to lack of satisfaction with food, these are integral components of OHQoL. Therefore, it is essential that supportive care is provided to patients to overcome these side effects. Artificial saliva (81), pilocarpine spray (56), local application of herbal products (82–84), close monitoring and individual coaching (57), may help to minimize radiation induced negative effects on OHQoL.

Both HNC and OPMD patients were reported with psychological symptoms such as low mood, anxiety and depression following diagnosis (30, 85). Poor OHQoL may be a predisposing factor for psychological distress (62). In addition, genetic links between psychological factors and quality of life in HNC patients have been identified (86). Psychological support and counselling is a critical need in HNC patients; the study by Nik and colleagues disclosed that psychosocial interventions enhanced post traumatic growth, and perceived spousal support reduced psychological complications such as depression, anxiety, and posttraumatic stress in HNC survivors (87).

In most of the studies included in the current systematic review, OHQoL assessment was conducted using the oral health impact profile short version (OHIP-14). Despite being a generic tool applicable for both healthy and disease populations, the ease of administration, coverage of seven domains, and cross-cultural adaptability has made it widely popular among researchers. A recent scoping review assessing different measures of OHQoL identified significant flows in the development and validation of tools used to measure OHQoL (88). Their study concludes that research reporting OHQoL in HNC may not comprehensively assess the full impact of the disease and its treatment on OHQoL, due to limitations and standardization issues in the assessment tools (88). Another scoping review therefore recommended using the Vanderbilt Head and Neck Symptom Survey version 2.0 (VHNSS 2.0) as it provides broader coverage of domains relevant to this patient group (89).

The findings of this systematic review should be interpreted with caution given the heterogeneity of the included studies, absence of uniform assessment measures and variation in the definition for OHQoL. Another limitation of the study, which resulted from the wide variation of the included studies, is the absence of quantitative data synthesis (e.g., meta-analysis) and temporal data analysis. This limits estimation of pooled effects and statistical interpretations, together with trends over time. Furthermore, a gray literature search was not conducted which could lead to a degree of bias.

## 5 Conclusions

OHQoL assessment is a critical need in HNC and OPMD patients and should be frequently monitored during the course of the disease, from the diagnosis stage, during the treatment and beyond. It is influenced by socioeconomic factors, body mass index, anatomical location of the malignancy, therapeutic modality, oral health status, reconstruction and rehabilitation methods. Compromised OHQoL can predispose to malnutrition, and psychological deterioration in patients. Nutritional assessment with dietary modifications, extended domestic and social support network, and psychological support are evidence-based recommendations to be incorporated into routine care pathways for HNC patients. Clinicians should be aware of the various factors related to HNC patients and how they influence OHQoL to devise tailored management protocols for individual patients. Increased awareness, together with the need for the modifications of determinants related to patient factors, diagnosis and treatment, and post treatment can help to improve the OHQoL of patients with HNC.

## Data Availability

The raw data supporting the conclusions of this article will be made available by the authors, without undue reservation.
