# Supplementary material for: Oral health–related quality of life in head and neck cancer: a systematic review
Source: Front Oral Health. 2025 Nov 19;6:1691065. doi: 10.3389/froh.2025.1691065 (PMC12672552; doi:10.3389/froh.2025.1691065)
Supplement: Supplementary file 1 [file Table1.docx]

Table S1: Search strings used for the electronic literature search in Databases

| Database | Search String | Number of studies | | | |
| --- | --- | --- | --- | --- | --- |
|  |  | Search 1 | Search 2 | Search 3 | Total |
| PsycINFO (Ovid) 1967 | (OHIP* OR OHIP OR “Oral health impact profile” OR “Oral health quality of life” OR OIDP* OR “Oral impact on daily performance”) AND (Cancer OR Precancer* OR Malig* OR Premalig* OR Neoplasms OR Tumor*) |  |  | 1 |  |
| EMBASE 1974 | 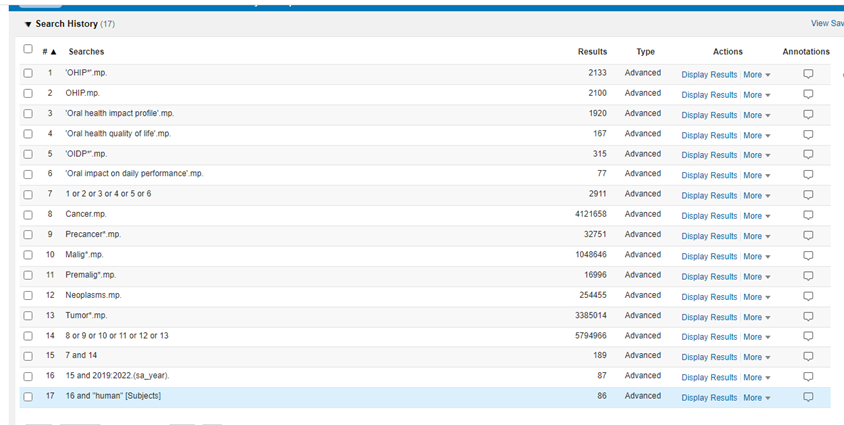 |  |  | 51 |  |
| OVID MEDLINE(R) 1946 | 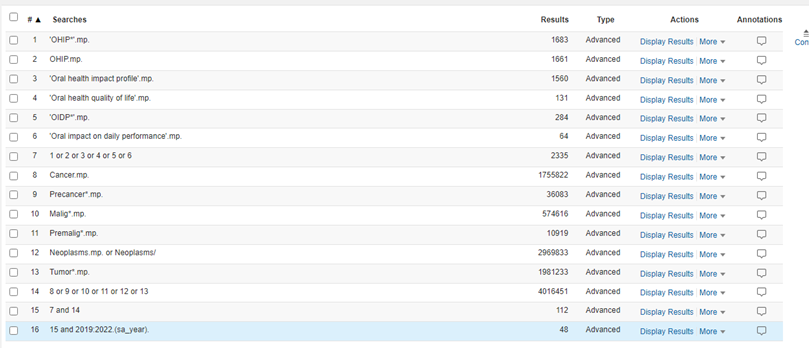 |  |  | 21 |  |
| SCOPUS | **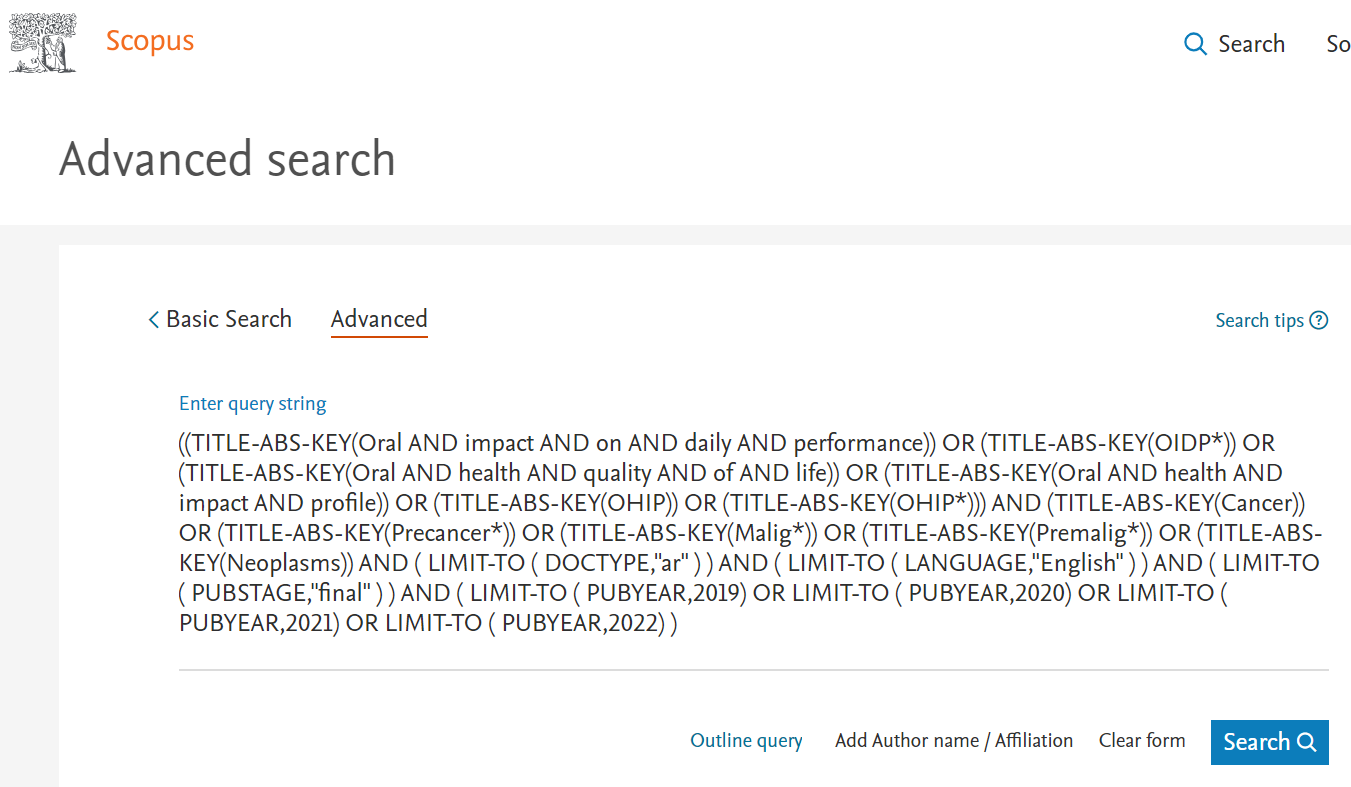** |  |  | 324 |  |
| WEB OF SCIENCE | 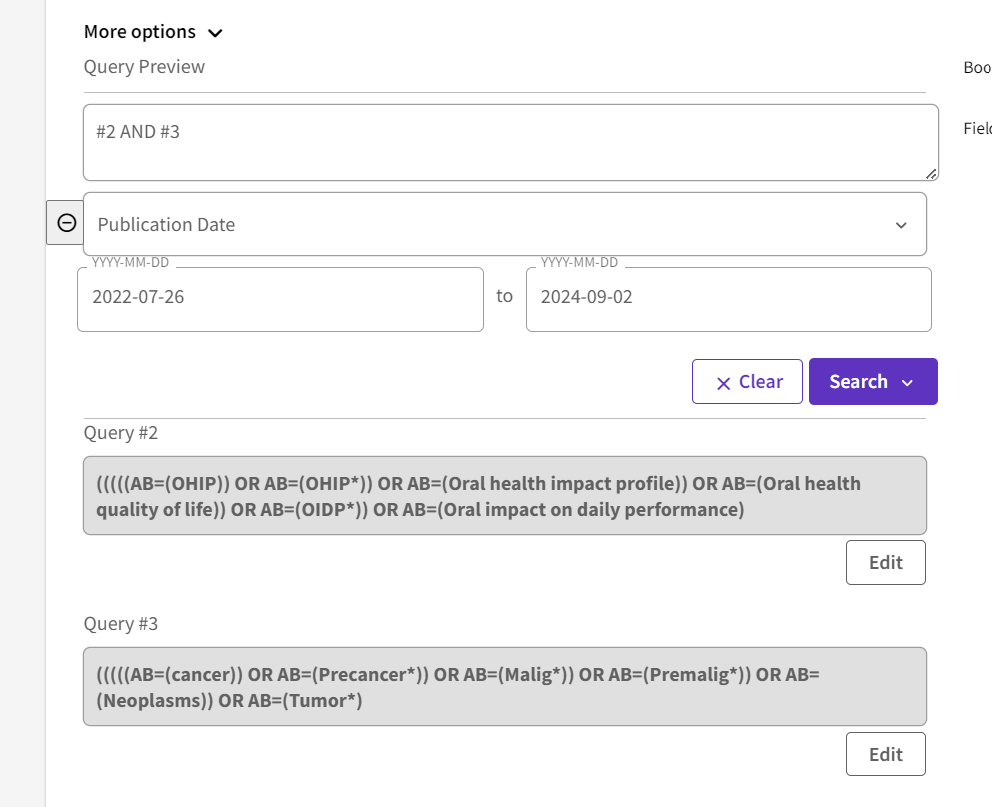 |  |  | 201 |  |
| Total | | 330 | 270 | 598 | 1196 |

Table S2: Studies on HNC patients included in the systematic data synthesis (n=93)

|  | **Author-Year** | **Anatomical location of lesion** | **Country** | **Time of OHQoL assessment** | **Sample**  **Size** | **OHQoL tool** | **Other**  **assessments** | **Main findings in relation to OHQoL** |
| --- | --- | --- | --- | --- | --- | --- | --- | --- |
| 1 | (Abed et al., 2023) | Tongue = 56.6%  Oropharynx = 26.6%  Larynx= 6.6%, Thyroid=6.6%  Paranasal sinuses = 3.3% | United Kingdom | Post radiotherapy | HNC=30 | OHIP-14 | Global self-ratings of general and oral health | - No association found between the length of the dental arch and OHQoL. - Length of dental arch alone does not explain why HNC patients have difficulty eating post-radiotherapy |
| 2 | (Andreassen et al., 2022) | Oral cavity = 35.2%, Oropharynx = 59.3%  Nasopharynx = 2.5%  Maxillary sinuses = 0.6%,  Salivary glands = 1.8% | Norway | Post treatment (>80% surgery and radiotherapy) | HNC = 162 | OIDP | - | - The HNC survivors had four times the risk of reporting problems with daily performances compared with the general population cohort. - The ability to eat and enjoy food was most frequently affected. - Moderate to poor self-rated dental health and general health as well as high frequency of dental visits were significantly associated with poorer OHQoL |
| 3 | (Aparna et al., 2022) | Head and neck | India | Post radiotherapy | HNC=150  controls = 150 | OHIP-14 | SF-12, WHO 2013 oral health proforma | - Oral health status, health related quality of life and OHQoL of HNC group was poor compared to control group. - Radiotherapy had a significant negative impact on OHQoL |
| 4 | (Barkokebas et al., 2015) | Nasopharynx = 3.3%  Langerhans cell histiocytosis Hodgkin’s lymphoma, Non-Hodgkin lymphoma AML Neuroblastoma Rhabdomyosarcoma Ewing sarcoma = 96.7% | Brazil | During radiotherapy | HNC=60 | OHIP-14 | - | - OHQoL is significantly affected by oral mucositis in individuals diagnosed with cancer during radiotherapy. |
| 5 | (Barrios et al., 2015a) | Tongue =35.2%  BM=12.7%  FOM, gingiva and oropharynx=11.3% each  Other = 18.3% | Spain | 6 months post treatment | OCA=142  Controls = 142 | OHIP-14, OIDP | SF-12 | - Oral cancer patients had worse OHQoL, worse physical HRQoL and similar psychological HRQoL than the general population. - The most important differences, both in OHIP-14 and OIDP, were found in items associated with eating. |
| 6 | (Barrios et al., 2014) | Tongue =47%  Gingiva =11.3%  FOM=11.3%  Oropharynx=13.5%  Retromolar =7.5%  Other=21.1% | Spain | 6 months post treatment | OCA=133 | OHIP-14, OIDP | NA | - Overall, 97%reported having at least one oral impact in the last month. - Eating was the most prevalent impact, followed by speaking. physical pain, functional limitation, and physical disability were the dimensions with the higher impact. - Being malnourished or at risk of malnutrition is an important longer-term determinant of worse OHQoL among patients treated for oral cancer. - Better OHQoL among males, older patients, and those with more   functional tooth units while patients that had undergone radiotherapy had significantly worse OHQoL |
| 7 | (Barrios et al., 2015b) | Tongue =35.2%  BM=12.7%  FOM, gingiva and oropharynx=11.3% each, Other = 18.3% | Spain | 6 months post treatment | OCA=142 | OHIP-14, OIDP | NA | There was a significant association between long-term OHRQoL and HRQoL in oral and oropharyngeal cancer patients. |
| 8 | (Binnal et al., 2021) | Oral cavity=81.5%  Oropharynx= 18.5% | India | At the time of diagnosis before treatment | OCA and OP cancer=108 | OHIP-14 | SF12V2 | - Older age, rural and poor socioeconomic background, substance abuse, systemic comorbidities and obesity were associated with poor OHRQoL. - Psychological issues were higher amongst younger participants. - Tobacco and alcohol consumption negatively impacted physical measures but had a masking effect on psychological parameters. - Patients who used tobacco reported higher oral health-related social disability than those who never used tobacco.A positive association between duration of SLT usage and physical pain was reported, where the pain scores increased with increase duration of SLT use - Daily users of tobacco and alcohol reported better OHQoL scores than occasional users - Pain was the single most significant negative determinant of OHRQoL that was associated with the worst OHIP scores. |
| 9 | (Brandão et al., 2016) | OSCC = 47.5%  Bone tumors of the mandible = 52.5% | Brazil | Post surgical management with mandibular reconstruction | OCA=40 | OHIP-14 | - | The surgical guides (intraoral and extraoral) used during ablative surgery proposed in this study directly improved anatomy, esthetics, and OHQoL after mandibular reconstruction. |
| 10 | (Čanković et al., 2022) | Oral cavity and/or pharynx = 44.9%  Larynx = 29%  Other =26.1% | Serbia | During and on completion of treatment | HNC=345 | OHIP-14  EORTC H&N35 | EORTC QLQ-C30, European Health Interview Survey | - Age influenced the OHIP score, education significantly affected it. - Employment/financial difficulties had impact on OHIP score. - Sexual problems influenced the OHIP score. - Patients with larynx and oropharyngeal cancers and patients who underwent radio/chemo therapy in addition to surgical treatment had higher OHIP scores. - Patients with gastrostomy and tracheostomy reported worse OHQoL. |
| 11 | (Coward et al., 2020) | Maxilla = 100% | United Kingdom | Following surgical resection | OSCC and bone tumors=19 | OHIP-49 | - | - All correlation coefficients were non-significant with regard to the responses to the OHIP-49 questions and the surface area and mean depth measurements restored by the obturators. - There is no simple relationship between the extent of facial change with and without an obturator and quality of life around appearance |
| 12 | (de Melo et al., 2018) | Oral cavity = 23.5% Pharynx =13.7% Larynx = 22.5% Neck region =15.7% Other = 24.5% | Brazil | During and after treatment | HNC=102 | OHIP-14 | SF-36 | - More than half the women diagnosed with cancer in an advanced stage had lower quality of life scores. - In addition, all those who underwent radiotherapy or chemotherapy had lower OHQoL |
| 13 | (de Melo et al., 2019) | Oral cavity= 23.8%  Pharynx= 16.2%  Larynx=23.8%  Cervical region=14.6%  Other = 21.5% | Brazil | Before or during treatment | HNC=130 | OHIP-14 | - | - Pain, physical disability and functional limitation were the main factors affecting the OHQoL. - non-Caucasians, widowers, patients diagnosed with squamous cell carcinoma and patients with pain the temporomandibular joint had higher impact on OHQoL with higher OHIP scores. |
| 14 | (Depeyre et al., 2020) | Tongue = 100% | France | Post surgery | 27 | Global Oral Health Assessment Index (GOHAI) | - | - In GOHAI, low score reflects poor OHQoL. All patients described a poor OHQoL, - Totally impaired patients were more affected than the partially impaired ones. - Impairment was related to food oral processing. - Study suggests that functional evaluation of food oral processing should be considered for patients treated for tongue cancer. |
| 15 | (Dholam et al., 2020) | Oral cavity = 75% Pharynx = 14%,  Larynx =10%,  Maxillary antrum = 1% | India | Before and after prosthetic rehabilitation | HNC=100 | LORQv3, OHIP-14 | - | - After 1 year of prosthetic rehabilitation there was improvement in OHQoL and in all domains of LORQ-v3 and OHIP. - For all items of the LORQ-v3 there was 10-38% improvement in function. OHIP-14 showed an 11 to 26% improvement in all domains. |
| 16 | (Dholam et al., 2016) | Palate = 34%  upper alveolus=19% BM=15%  Tongue =13% | India | Before and after prosthetic rehabilitation | OCA=75 | LORQv3OHIP-14 | - | - Although the scores were worse for radiated patient before rehabilitation. - There were no statistical differences seen between radiated and non-radiated group after 1 year of rehabilitation |
| 17 | (Dholam et al., 2017) | Oral cavity = 50% Oropharynx = 50% | India | Before and after prosthetic rehabilitation | OCA and OP cancers= 60 | LORQv3OHIP-14 |  | - In the OHIP-14 questionnaire, participants scored better after prosthetic intervention. - The highest percentages of change were noticed in the psychological disability and handicap domains. - Prosthetic rehabilitation contributed to an improvement in patients OHQoL |
| 18 | (Ettl, 2016) | Anterior - FOM, tongue, mandible = 43%  Lateral - FOM, base of the tongue, dorsal maxilla, oropharynx = 52%  Larynx and hypopharynx = 7% | Germany | One year after prosthetic rehabilitation | HNC=29 | OHIP-14  EORTC H&N35 | EORTC QLQ-C30, | - One year after denture placement, patients described significant improvement in the most relevant categories such as swallowing, social eating, social contact, dry mouth, and weight gain. - Xerostomia and implant insertion in the target volume of the irradiation are major reasons for implant failure, particularly for increased marginal bone resorption. - Implant insertion outside the target volume offers a very favorable prognosis comparable to that in non-irradiated patients. - Implant-based prosthetic rehabilitation improves patients' self-confidence, social integration, and OHQoL. |
| 19 | (Fromm et al., 2019) | Oral cavity = 61.2%  Hypopharynx = 11.1%  Oropharynx = 11.1%  Other= 16.6% | Denmark | Post treatment | HNC=18  controls=18 | OHIP‐49 | - | - OHQoL impaired in HNC patients compared to controls after oral rehabilitation. - The overall aesthetics was not significantly different between the two groups. - Within the HNC group, radiation therapy negatively influenced on the functional outcome after oral rehabilitation. - Impairment in OHRQoL is primarily caused by problems with speech and eating. |
| 20 | (Gondivkar et al., 2021a) | Buccal mucosa= 24%  Gingivo buccal sulcus= 43%  Tongue = 14%  Retromolar= 9%  Labial mucosa=5%  Palate=4%  FOM=1% | India | Post treatment | OCA=100 | OHIP-14 | - | - The overall OHIP-14 scores indicated that patients with trismus reported greater impairment of OHRQoL than those without trismus at the end of treatment and 3 months follow-up. - Patients with trismus suffer greater impairment of OHQoL. |
| 21 | (Gondivkar et al., 2021b) | Gingivo-buccal sulcus= 41.5%  Buccal mucosa= 22%  Tongue= 17%  Retromolar region=11.9% | India | Post treatment | OCA=135 | OHIP-14 | SF-12 | Irrespective of the post-treatment duration, patients who received post-surgical chemo-radiation had worse OHQoL compared to surgery only and post-surgical radiation groups. |
| 22 | (Hagio et al., 2018) | Maxilla = 50%  Mandible = 34%  FOM = 6%  Tongue= 10% | Japan | Pre and post treatment | OCA=50 | OHIP-J54 Japanese version | - | - The OHQoL of participants was improved in both the maxillary defect group and the mandibular region defect group. - Participant sex and functions including masticatory function, swallowing function, and articulatory function were associated with maxillofacial prosthetic placement, leading to improved OHQoL. |
| 23 | (Hassel et al., 2012) | Mandible = 100% | Germany | Post treatment | OCA=24 | OHIP-G49 German version | NA | Compromised OHQoL predicts psychological outcomes, namely depression and anxiety, better than vice versa. |
| 24 | (Huang et al., 2021) | Lip =2.7%  Buccal mucosa =34.5%  Tongue = 37.2% Gingivae = 16.9%  FOM=4.1%  Hard palate = 4.1% Retromolar = 0.7% | Taiwan | Post  Treatment | OCA=148 | OHIP | Self-care behaviors | - Patients who had more severity of adverse effects after self-care behaviors, who used feeding tubes during treatment, and who had BMI less than 25 were more likely to have worse overall OHQoL. - Psychological disability, functional limitation, and physical pain are the strongest factors affecting OHQoL. |
| 25 | (Indrapriyadharshini et al., 2017) | Buccal mucosa = 62.2%  Alveolus = 14.4%  Tongue= 14.4%  Other= 9% | India | Post treatment | OCA=90 | OHIP-14 Tamil  Version |  | - OHQoL was better in the surgery alone group compared to combined treatment modalities. - The “functional limitation” “physical pain” and “physical disability” domains were found to be more affected - Participants in the surgery, radiotherapy, and chemotherapy groups were found be affected more. |
| 26 | (Ishida et al., 2015) | Mandible = 100% | Japan | Post treatment (3 months after oral rehabilitation) | Malignant and benign tumors=26 | OHIP-49 Japanese version modified Sato questionnaire | NA | - Reconstruction with an osteo-cutaneous flap to build mandibular continuity produced good results in physical and eating ability after mandibulectomy, therefore recommended as the first-choice procedure - The missing continuity of the mandible reconstructed with soft tissue alone caused muscle fatigue and a limitation in lateral mandibular movement. - If a sufficient major flap was used for the reconstruction in mandibulectomy cases, missing mandibular continuity had the advantage of a greater maximum mouth opening range. |
| 27 | (Jung et al., 2019) | Head=75.6%  Neck = 24.4% | Korea | Post treatment | HNC=50 | OHIP-14 | - | - Patients who developed mucositis during radiotherapy had lower OHQoL than those who did not. - However, there was no difference in oral health status according to mucositis |
| 28 | (Kalaignan and Shree Mohan, 2018) | Mandible = 100% | India | Post treatment (prosthetic rehabilitation) | OCA=20 | OHIP-EDENT | maxillofacial prosthesis performance scale  obturator functioning scale | - The most prevalent impact on OHQOL was by psychological discomfort - More than half the percentage of patients upset with dental problems and self-conscious about the prosthesis after the 2 weeks of prosthesis function. - Despite the discomfort in psychological subscale, there was significant improvement in functional limitation, physical pain, psychological disability and social disabilities after 2 weeks of prosthesis. - When comparing to 2 weeks, all subscale scores showed significant progress on OHQoL after 3 months |
| 29 | (Karayazgan-Saracoglu et al., 2017) | Mandible = 100% | Turkey | Post treatment ( 5-year disease free survival and prosthetic rehabilitation) | OCA=22 | OHIP-EDENT | Visual analogue scale for patient satisfaction | - Both overdenture and fixed metal-acrylic resin prostheses can be acceptable treatment alternatives in relation to patients’ expectations. - OHIP-Edent values in the group with an overdenture prosthesis were higher than those in the group with a fixed metal-acrylic resin prosthesis - An implant-retained fixed metal-acrylic resin prostheses can be considered a more predictable treatment option for improving OHQoL in patients with marginal mandibular defects. |
| 30 | (Kumar et al., 2016) | Mandible = 100% | India | Post surgery, before and after prosthetic rehabilitation | Malignant tumors, benign tumors and osteomyalitis=46 | OHIP-14, Overall denture satisfaction index (DSI) | EORTC QLQ-C30, EORTC H&N35 | - Implant-supported removable overdentures improve OHQoL outcomes in patients with reconstructed mandibles. - This study showed no significant difference in QoL outcomes in patients with two- or four-implant supported removable prostheses. |
| 31 | (Li et al., 2017) | Maxilla= 55.8%  Mandible= 44.2% | Japan | More than three years of disease free survival with prosthesis | Malignant and benign=77 | OHIP-14 | SF-36 | - High prevalence of poor sleep quality was identified in long-term head and neck cancer survivors. - Extensive neck dissection, poor mental health, and psychological disability may contribute to poor sleep quality. - Maintaining good OHQoL could promote better sleep in these patients. |
| 32 | (Li et al., 2013) | Tongue =51%  FOM=28%  Gum =12%  Buccal mucosa=6%  Palate =4% | China | Post surgery with flap reconstruction | OCA=51 | OHIP-14 Chinese version | UW-QoL  Chinese version | - In the UW-QoL the best-scoring domain was pain, whereas the lowest scores were for chewing, saliva, and taste. - In the OHIP-14 the lowest-scoring domain was handicap, followed by psychological disability, and social disability. - Patients with tongue and floor of the mouth malignancies reported a poorer OHQoL outcome than those with malignancies in the buccal mucosa |
| 33 | (Li et al., 2016) | Tongue= 73.17%  Tongue and FOM =26.83% | China | Post operative | OCA=41 | OHIP-14 | UW-QoL | - Using either Radial free forearm flap (RFFF) versus pectoralis major pedicled flap (PMMF) for reconstruction of defects after tongue cancer resection significantly influences a patient’s OHQoL. - significant difference between the psychological discomfort and social disability components of the OHIP-14 questionnaire was evident between the 2 groups. |
| 34 | (Linsen et al., 2009) | Maxilla= 53.8%  Mandible= 46.2% | Germany | Post treatment | OCA=26 | OHIP-G-53 German version | - | - This study reveals significant differences in the physical pain domain. - Patients with resections of the maxillary had lower OHQoL compared to mandibular defects. |
| 35 | (Maeda et al., 2018) | Gingival cancer=90.4%  Intra osseous carcinoma= 6.4%  Ameloblastoma=3.2% | Japan | Post treatment | 31  Mandibular angle and mental tubercle (AT group) = 7  Mandibular Body (body group) =12  Marginal resection (MR group) = 12 | OHIP-49 | - | - Body group scored significantly higher than the AT group in the OHIP. - There was a significant difference between the AT group and body group in the physical disability and psychological disability domains. - The body group showed higher stomatognathic performance than the AT group both subjectively and objectively, but there was little difference in the chewing performance |
| 36 | (Malouf et al., 2003) | Head and neck | USA | Pre and post radiotherapy | HNC=93 | Xerostomia-related quality of life scale | SF-36 | - Unilateral and bilateral neck RT with parotid-sparing techniques were successful in preserving salivary output, compared to standard three beam RT techniques. - Lower radiation dose to contralateral parotid glands was associated with greater percentage of baseline salivary flow rates at 1-year post-RT, fewer xerostomia complaints, and an enhanced OHQoL |
| 37 | (Maqbool et al., 2021) | labial mucosa=4.6%  Buccal mucosa=3.4%  Tongue=36%  gingiva=14% FOM=6.9%  soft palate=8%  hard palate=3.4%; tonsil=4.6%  paranasal sinuses and nasal cavity=4%;  salivary  glands =4.6% pharynx=10.5% | Pakistan | During radiotherapy | 150 patients, 150 controls | OHIP-14 | SF-12 | - Radiotherapy has a serious negative effect on OHQoL. - The mean OHIP-14 scores were significantly higher among head and neck cancer group compared to control group |
| 38 | (Martins et al., 2021) | Oral cavity = 25%  Base of tongue = 54.2% Rhinopharynx=2.1% Hypopharynx= 10.4%  Glottis and supraglottis= 8.3% | Brazil | During radiotherapy | Photobio-modualtion therapy group = 25, control group = 23 | OHIP-14 Brazilian version | - | - Findings showed that radiotherapy and oral mucositis is associated with impairment in patients OHQoL. - Photobiomodualtion therapy was effective in preventing the impacts in OHQoL, - Mainly in the final stage of RT and reduce the mucositis related symptoms reported by patients. |
| 39 | (McMillan et al., 2004) | Nasopharynx = 100% | Hong Kong | Newly diagnosed and post treatment | survivors=38  new patients=41 controls=31 | OHIP-49 Cantonese version | SF‐36 | - There were significant differences in the functional limitation, physical disability, social disability and handicap domains, with a higher mean score among survivors. - There were differences in summary scores between groups indicating a greater overall negative impact on OHQoL in survivors compared with new patients and controls. |
| 40 | (Mertens et al., 2016) | Maxilla = 100% | Germeny | Pre and post treatment | OCA=5 | OHIP-EDENT | - | - The CAD/CAM supra-structures improved the OHQoL. - Significant improvement concerning general satisfaction, stability, ability to chew, esthetics, pain, speech, and self-confidence. |
| 41 | (Naidu et al., 2019) | Head and neck | India | During treatment | HNC=50 | OHIP-14 | - | - The findings showed more impact of chemotherapy and combination therapy on the OHQoL. - Higher incidence of functional and physical disability in males than females. |
| 42 | (Nascimento et al., 2019) | Oropharynx =40.0%  Oral cavity = 32.5% Larynx = 10.0% Nasopharynx = 7.5%  Others =10.0% | Brazil | Post treatment | HNC=40 | OHIP-14 | - | xerostomia has a negative impact on the OHQoL of patients who undergo radiotherapy |
| 43 | (N. F. Pereira et al., 2020) | Head and neck | Brazil | Post treatment | Pilocarpine=20  Placebo=20 | OHIP-14 Brazilian version | - | The topical application of pilocarpine spray was similar to placebo on stimulated whole salivary flow assessment and OHQoL scores in patients treated with radiotherapy for HNC |
| 44 | (Pieralli et al., 2021) | Mandible and anterior FOM=70.2%  Maxilla=5.4%  Oropharynx = 16.2% | Germany | Post treatment | HNC=37  Controls=20 | OHIP-G14 German version | Patient reported outcome measures - PROM | - The OHQoL of irradiated patients were lower compared to the other cohorts. - OHQoL were not influenced by sex or type of prosthetic treatment. - Poor OHQoL in the HNC group who had tumor resection with adjuvant radiotherapy compared to HNC group that had tumor resection only and the control group. |
| 45 | (Pow et al., 2012) | Nasopharynx = 100% | Hong Kong | Post treatment | HNC=58 | OHIP-49 | SF-36 | - Intensity Modulated Radiation Therapy (IMRT) for early-stage nasopharyngeal carcinoma could only partially preserve the whole salivary function and OHQoL. - The parotid saliva flow had recovered fully after 1 year and whole saliva flow had recovered to 40% of baseline. - A general trend of deterioration OHQoL was observed after IMRT, followed by a gradual recovery. Persistent oral-related symptoms were found 2 years after treatment. |
| 46 | (Said et al., 2017) | Maxilla = 100% | Japan | Post treatment | OCA=38 | Geriatric Oral Health Assessment Index (GOHAI) | - | - The type of defect closed or open, in patients with a partial maxillectomy may not influence their masticatory function and overall OHQoL if they have had adequate prosthetic rehabilitation. - Special consideration should be given to patients with an open defect with regard to swallowing function and psychological concerns in order to improve their OHQoL. |
| 47 | (Santos et al., 2017) | Tonsil palate = 23.3%  Tongue =13.3%  Buccal floor =13.3% Gum = 10%  Nasopharynx=10%  Larynx = 10%  Cheek mucosa = 6.6%  Vocal cords = 3.3%  Adenoid = 3.3%  Lips = 3.3%  Hypophysis = 3.3% | Brazil | Post treatment | HNC=30  Control=45 | OHIP-14 | NA | Caries activity, periodontal disease index and incidence of edentulism were high and OHQoL was compromised in individuals undergoing radiotherapy for HNC compared to controls. |
| 48 | (Schweyen et al., 2017) | Nasopharynx = 5.2%, Oropharynx = 26.7%, Uvula= 0.9%  Tongue base = 5.2%  Oral cavity = 32.8%  Parotid gland = 7.8%, hypopharynx/  larynx = 21.6% | Germany | Post treatment | HNC=116 | OHIP-G14 German version | - | - The average values of none, fixed partial dentures and removable partial denture patients were higher than those found in a normal population, but did not differ significantly from each other. - The highest mean score was found for patients with an oral cavity tumor site, while cancers located in the nasopharynx had the lowest value. - Average OHIP scores for patients irradiated with IMRT did not differ significantly from those in patients irradiated with 3D-CRT |
| 49 | (Soldera et al., 2020) | oral cavity and oropharynx = 60%  hypopharynx and larynx= 40% | Brazil | Post treatment | HNC=90 | OHIP-14 | - | - The OHQoL of survivors of HNC experienced a negative impact following radiotherapy. - The impact was associated with hyposalivation and advanced stage tumors. - The highest scores were found for the functional limitation followed by the physical pain. |
| 50 | (Stefano et al., 2019) | Tongue = 100% | Italy | Post treatment, | OCA=6 | OHIP-14 | - | - Prosthetic rehabilitation incurred a clear improvement in the comfort, chewing, aesthetics and functionality of the patients - Results indicate an increase in overall satisfaction and self-esteem, re-established serenity of mind and a remarkable improvement in OHQoL - Palatal augmentation prosthesis is an effective therapeutic remedy to improve OHQoL in patients with absent or reduced lingual mobility following surgery |
| 51 | (Stuani et al., 2018) | Oral cavity= 54%  Parotid = 2.5%  Pharynx= 32.5%  Larynx = 12.5% | Brazil | At the time of diagnosis and post treatment | Before treatment=20  After treatment=20 | OHIP-14 | - | - Statistically higher difference in functional limitation and physical pain in post treatment group compared to newly diagnosed. - Patients already undergoing oncologic treatment also had a higher negative impact on OHQoL on functional limitation dimension than those that did not start the treatment |
| 52 | (Tesic et al., 2020) | Larynx = 29%  Oral cavity and/or pharynx (epi-, oro-, hypo-pharynx) = 44.9%  Other= 26.1% | Serbia | Post treatment | HNC=345 | OHIP-14  EORTC H&N35 | EORTC QLQ-C30, | - Better OHQoL in patients who had only surgery compared to those who had surgery accompanied with radio- and chemotherapy. - Patients with a tumor stage 0-II also had better OHQoL compared to those who had a tumor stage III-IV. - The OHIP-14 correlated significantly with the QLQ-C30 and QLQ-H&N35 scales. |
| 53 | (Wang et al., 2019) | Buccal = 15.25%  Tongue =47.46%  FOM=16.95%  Gum=5.08%  Parotid gland =3.39%  Pharynx = 6.78%  Other = 5.07% | China | Post treatment | HNC=59 | OHIP-14 | UW-QoL | - Three flaps namely radial forearm free flap (RFFF), ulnar forearm free flap (UFFF), and anterolateral thigh flap (ALTF) were compared for OHQoL outcomes. - ALTF group had significantly lower social disability scores than the RFFF and UFFF. - There were no significant differences for other indicators. |
| 54 | (Westgaard et al., 2021) | Oropharynx =25.4%  Oral cavity = 10.2%  Salivary gland = 8.4%  Nasopharynx= 1.7%  Unknown origin= 3.4% | Norway | Post treatment | HNC=59 | OHIP-14 | - | The study demonstrates that HNC patients treated with intensity-modulated radiotherapy still experience late effects in terms of xerostomia and subjective ocular dryness, and these have a clear negative impact on their OHQoL. |
| 55 | (Winter et al., 2021) | Head and neck | Germany | Post treatment | HNC=52 | LORQv3, OHIP-14, EORTC QLQ-OH15 | Satisfaction with Life Scale (SWLS) | OHQoL was impaired in HNC patients in general, yet we found a particularly high need for supporting OHQoL among the subgroup of patients with the need for oral rehabilitation and after multimodal therapy. |
| 56 | (Witsell et al., 2012) | Oral cavity/Tongue =29.6%  Oropharynx= 29.6%  Salivary gland= 12.9%  Hypopharynx = 3.7%  Larynx =5.5%  Other = 11.1% | United Kingdom | Post treatment | Cevimeline= 28  Placebo=26 | OHIP-49 | UW-QoL | - Grade of xerostomia decreased (improved) for both cevimeline-treated and placebo-treated patients over the course of the study. - No statistically significant differences in OHQoL or QOL were observed. |
| 57 | (Wolff, et al., 2004) | maxilla = 50%  tongue = 16.6%  oral cavity not specified = 16.6%  left tear duct = 16.6% | Canada | Post treatment | OCA=6 | Psychosocial Aspects of Prosthetic Use Scale (PAPUS) | - | Overall, the results using the PAPUS with participants suggest that the participants were satisfied with their prostheses and that this satisfaction positively impacts OHQoL. |
| 58 | (Xiao et al., 2019) | Oral cavity (buccal, maxilla, or palate) = 48%, Spaces (infratemporal, ptygo-palatine and parapharyngeal) = 16 %  Nasal cavity or paranasal sinus =8% Parotid or periauricular region = 28% | China | Post treatment | 25 | Skull base inventory (SBI) | NA | Anterior skull base (ASB) involved cases in our cohort were more likely to have better long-term OHQoL than patients who had mid skull base (MSB) involved tumors. |
| 59 | (Xu et al., 2022) | Tongue = 47.9%  FOM=31.3%  Buccal mucosa =12.5% | China | Post treatment | RFFF group = 30  GSFF group = 18 | OHIP-14 | Vancouver Scar Scale (VSS), UW-QoL | - Compared with patients who under-went reconstruction with a radial forearm free flap (RFFF), patients reconstructed with groin soft tissue free flap (GSFF) exhibited a better appearance of the donor site and better mood, psychological status, OHQoL and social relationships. - The OHIP-14 scores for psychological discomfort and social disability differed significantly between the RFFF and GSFF groups. - The overall average domain score of the OHIP-14 in the GSFF group was lower than that in the RFFF group; however, the difference was not statistically significant |
| 60 | (Yanamoto et al., 2020) | Maxilla= 100% | Japan | Post treatment | OCA=5 | OHIP-J49 Japanese version | - | - This study indicated that an obturator relined with soft silicone improved masticatory performance and the OHQoL post-maxillectomy. - Functional limitations domain indicated a statistically significant improvement between the acrylic resin and silicone soft reliner obturator prosthesis. - The pain domain was not statistically significant, but tended to improve after the application of the silicone soft reliner. |
| 61 | (Yang et al., 2014) | Alveolus = 38.23% FOM= 26.47%  Buccal mucosa = 20.59%  Tongue =14.71% | China | Post treatment | 34 | OHIP-14 | UW-QoL | - In present study, for UW-QOL scale the best-scoring domain was mood, and the worst score of the domains are chewing and saliva. - The selection of the most important three domains, chewing was considered most important followed by speech and swallowing. - In the OHIP scale the best domain was for social disability, handicap, and psychological disability. - The highest score was for physical disability and physical pain. |
| 62 | (Yuan et al., 2016) | Tongue = 100% | China | Post treatment | 67 | OHIP-14  EORTC H&N35 | EORTC QLQ-C30, | - Free anterolateral thigh perforator flap (FATPF) was compared with Free vascularized forearm flap (FVFF) for reconstruction of tongue. - Functional scores decreased over time after the procedure (6-12 months after surgery) and there were significant differences between the 2 time points for all domains. However, there were no relevant differences between the 2 flap types for all domains at 12 months. - Psychological disability showed the lowest scores and the highest scores were for physical disability. |
| 63 | (Yusa et al., 2017) | Mandible = 100% | Japan | Post treatment | Malignant and non-malignant disease =12 | OHIP-14 | - | - Prosthetic rehabilitation improved masticatory function. - In nonmalignant cases, there were significant improvement in the domain of functional limitation. - Functional limitation, psychological discomfort, physical disability and psychological disability were significantly improved in malignant cases. |
| 64 | (Zhang et al., 2020) | Tongue= 100% | China | Post treatment | OCA=65 | OHIP-14 | UW-QoL  SF-36 | - Free anterolateral thigh perforator flaps for reconstruction of glossectomy defects after cancer resection have significantly influenced the patients OHQoL. - The best domain scores was for handicap, psychological disability, and social disability. The highest scores were for psychological disability and physical pain. |
| 65 | (Zhu et al., 2017) | Tongue = 100% | China | Post treatment | HNC=32  Controls=20 | OHIP‐49 Chinese version | - | - Radial forearm free flap (RFFFs), anterolateral thigh free flaps (ALTFFs) and nasolabial island flap (NLIFs) were compared. - The scores of psychological discomfort of RFFFs and ALTFFs were significantly higher than NLIFs - OHQoL were better in patients undergoing NLIFs reconstruction surgery. |
| 66 | (Zhu et al., 2021) | Tongue= 100% | China | Post treatment | OCA=20  Controls=20 | OHIP-49 | - | - All dimensions of the OHIP-49 at 9 months and 18 months were significantly higher compared to controls except for physical discomfort domain. - Moreover, OHIP-49 scores were significantly lower for elderly patients (age ≥55 years) than those of younger patients (age <55 years) indicating that younger patients had a better OHQoL after surgery. - Physical disability, handicap scores were significantly higher in men than in women. - psychological discomfort, social disability, handicap and total scores in patients who received postoperative chemotherapy were significantly higher than those in patients who did not. - Scores for handicap in patients who received postoperative radiotherapy were significantly higher than patients who did not. - Somatosensory disturbances observed after surgery were associated with poor OHQoL. |
| 67 | (Chouksey et al., 2023) | Maxilla = 100% | India | Post treatment | Cancer= 47  Mucormycoses = 32 | OHIP-14 |  | - Comparison of the OHRQoL after rehabilitation with prostheses among patients with cancer and post COVID-19 associated mucormycosis - OHRQoL scores was much higher among patients with mucormycosis before rehabilitation (compared with patients with cancer), were almost similar after rehabilitation |
| 68 | (Philipp Jehn et al., 2024) | Maxilla= 50%  Mandible=50% | Germany | Post treatment | OCA=8 | OHIP-G53 | - | - Total OHIP scores of all patients after rehabilitation with dental implants indicated acceptable OHRQoL. - Worse OHRQoL was reported in patients who had lower jaw treatment compared to upper jaw |
| 69 | (Shirakawa et al., 2024) | Tongue = 94.2%  Tongue and FOM= 5.8% | Japan | Post treatment | Partial glossectomy= 11  Hemi glossectomy=16  Total glossectomy=8 | OHIP-14 | - | - Following glossectomy, tongue pressure correlated with the overall OHIP-14 score, and with psychological and social disability domain scores. - physical disability was correlated with swallowing function (RSST) - social disability was significantly correlated with Oral Diadochokinesis ODK, tongue pressure and RSST. - tongue pressure was significantly correlated with social disability, exhibiting a negative correlation. - Our study suggests that assessing tongue pressure, a key aspect of postoperative oral function, is valuable not only as an indicator for predicting a decline in postoperative OHQoL but also as a target for rehabilitation that may effectively prevent a decline in postoperative OHQoL. |
| 70 | (Ghorbani et al., 2023) | Tongue = 58.8% Mandible = 9.8% Buccal mucosa = 9.8%  Mouth floor =7.8% Lip =7.8% Locoregional metastasis = 5.9% | Iran | Post treatment | OCA=51 controls=51 | OHIP-14 | - | - All aspects of OHIP, patients scored higher than the control group - This difference was only significant in functional limitation and physical pain domains. - Among the existing treatments, surgery had the lowest and combination therapy had the highest reduction in OHQoL. |
| 71 | (Martins et al., 2021) | Oral cavity = 7.7%  Hard palate = 7.7%  Tongue =21.2%  FOM = 9.6%  Base of tongue=44.2% Rhinopharynx = 3.8%  Glottis and supra glottis = 3.8%  Occult primary = 1.9% | Brazil | During treatment | Photobiomodulation group = 27  Preventive Oral Care Program group=25 | OHIP-14  Patient-reported oral mucositis symptom scale. | UW-QoL | - The effect of mucoadhesive phytomedicine (Curcuma longa L. and Bidens pilosa L) on radiotherapy induced oral mucositis was assessed. - Both groups experienced worsening of OHQoL. - There was no statistically significant variation of OHQoL in relation to the two treatment methods |
| 72 | (Caminha et al., 2024) | Tongue base= 83 %  Oropharynx=8.33%  Larynx = 8.33 % Nasopharynx = 8.33 %  Tongue mouth floor = 4.16 %  Pharyngeal tonsil=4.16 % Hypopharynx=4.16 %  Alveolar ridge=4.16%  Lower lip =4.16 % Tonsillar fossa =4.16 % | Brazil | Post treatment | HNC=24 | OHIP-14 | - | - RT and oral mucositis worsened OHQoL in patients. - Physical pain, functional limitation, and physical disability were the dimensions with the higher impact. - Radiotherapy significantly associated with functional and physical pain domains. |
| 73 | (Winter et al., 2023) | Head and neck cancer with metastasis or recurrence | Germany | Post treatment | 21 | LORQv3 | Geriatric assessment, Hospital Anxiety and Depression Scale (HADS), QLQ-C30, ELD-14 | - High impairments were found for the domains of oral function and orofacial appearance, indicating a low ability of chewing, swallowing, and mouth opening. - In addition to the side effects of surgical treatment and radio-/chemotherapy (xerostomia, restricted agility of oral structures, and facial disfigurement), the development of a recurrence/metastatic disease seems to cause an additional deterioration of OHRQoL. - The high incidence of frailty in patients suffering has a negative impact on the ability for oral rehabilitation. - Also had a higher psychological burden. |
| 74 | (Aguiar et al., 2024) | Larynx =39.1%  Oral cavity =47.85 Parotid =13.0% | Brazil | During radiotherapy | HNC=23 | OHIP-14 | Salivary flow rate | salivary flow rate reduced and OHQoL worsened during the course of the RT with a statistically significant difference. |
| 75 | (Afzal et al., 2023.) | Lips =3.8%  Tongue anterior two third =33.8% Mucosa of buccal cavity = 27.5%  FOM= 8.8%  Hard palate=10.0% Alveolar ridge =10 %  Retromolar trigone =2.5%  More than two sites =3.8% | Pakistan | Post treatment | HNC=81 | EORTC QLQ-OH15 | - | - The OHQoL of oral cancer patients tends to decline during the course of radiation therapy (RT), whether it's administered alone or in combination with chemotherapy. - OHQOL tends to improve three months after the completion of RT. |
| 76 | (Zeman-Kuhnert et al., 2024) | Mandible = 76.1%  Maxilla = 23.9% | Austria | Post treatment | HNC=113 | OHIP-49 | SF-13 | - patients showed slightly worse OHQoL after jaw reconstruction and dental rehabilitation than the standardized normal population. - Among the dental rehabilitated patients, OHQoL was best in patient with implant-supported prostheses |
| 77 | (Jiang et al., 2024) | Nasopharynx = 40.2%  Larynx = 14.1%  Hypopharynx = 9.7%  Oropharynx = 15.2%  Oral cavity = 20.6% | China | During radiotherapy | Intervention group= 47 control group = 45 | OHIP-14 | WHO oral health questionnaire for adults | - The mean score of OHIP-14 total was highest at the end of RT in both groups. - A good recovery of OHQoL was observed after 3 and 12 months in both groups. - Participants in the intervention group had a better out come over time in all scales compared to the control group - A statistically significant better OHQoL and subscales of physical pain, physical disability and psychological disability. - The integrated supportive programme showed positive effects on improving OHQoL regarding plaque control, the state of teeth and gums. |
| 78 | (Al-Aroomi et al., 2024) | Tongue = 88.6%  FOM= 20.7%  Buccal = 26.4%  Retromolar area = 7.5%  Oropharynx = 3.7% | China | Post treatment | Radial forearm free flap (RFFF) = 40  Ulnar forearm free flap (UFFF) = 40 | OHIP-14 | UW-QoL | UFFF exhibited a better appearance, social domain, and low decision regret compared with RFFF, indicating that the UFFF may contribute to improving postoperative OHQoL. |
| 79 | (Qayyum et al., 2023) | Buccal vestibule = 66.6%  FOM=11.1%  Lip= 5.5%  palatal mucosa = 5.5%  Tongue = 5.5%, Retromolar trigone = 5.5% | Pakistan | Before and after treatment | 18 | OHIP-14 | - | - The total OHIP-14 scores for patients before surgery, after surgery, and after dental rehabilitation were reduced and OHQoL improved. - Functional limitation and psychological discomfort improved after rehabilitation and surgery. - Physical disability in patients improved after dental rehabilitation along with social disability and handicap. - The OHQoL was markedly improved after the reconstruction of mandibular defects with free vascularized fibular flaps and dental rehabilitation. |
| 80 | (I Rodrigues et al., 2023) | Oral cavity | Portugal | Before treatment | OCA=46 | OHIP-14 | Oral health values scale | - As for the OHQoL there was no statistically significant differences were found between men and women. - More than a half of the sample were psychologically affected. - Comparing the self-perception of periodontitis with OHIP-14 domains, there were statistically significant findings observed concerning functional limitation and physical pain. |
| 81 | (N Matapathi et al., 2022) | Maxilla = 73.4%  Mandible= 26.6% | India | Post treatment | OCA=15 | OHIP-14 | chewing ability questionnaire, dysphagia score, dysarthria  assessment | - The mean OHIP-14 after prosthetic rehabilitation reduced significantly compared to the prescore suggesting an improvement in the OHQoL. - Statistically significant differences were observed for functional limitation, physical pain, psychological discomfort, physical disability, psychological disability, and handicap, - Except for social disability which showed no statistically significant differences. |
| 82 | (Reichal and Prethipa, 2024) | Buccal mucosa =33.33%  Lower alveolus = 29.73%  Tongue = 28.83%  Upper alveolus = 6.31%).  upper and lower alveolus = 0.9% FOM = 0.9% | India | Post treatment | OCA=111 | OHIP-14 | - | - The majority of cases reported were found to be at T4a tumor staging with a habitual duration of more than five years, and more than half of the study population had severe compromise in their OHQL. - The presence of perineural invasion and lympho-vascular invasion has an impact on nodal metastasis, treatment choices, recurrence, and OHQoL. |
| 83 | (Qamar et al., 2024) | Head and Neck | Pakistan | Post treatment | HNC=79 | EORTC QLQ-H&N-35 | Oral Hygiene Index, WHO- Oral mucositis scale | - Difficulty in mouth opening and dry mouth were main concerns related to OHQoL. - BMI, poor oral hygiene status, monthly income, oral mucositis grade were significantly associated with the decline in OHQoL within one year, post cancer treatment. - Lower mean score indicating better OHQoL in patients with moderate to severe oral mucositis using fluoride toothpaste compared to patients with mild oral mucositis who were not using fluoride tooth paste - Married patients reported better OHQoL and mouth opening capacity compared to patients who were not married |
| 84 | (Andreassen and Hadler-Oslen, 2022) | Oral cavity or pharyngeal cancer = 100% | Norway | Post treatment | OCA and OP cancers =162 | OIDP | - | - Reduced ability to eat and enjoy food was most strongly associated with being diagnosed with cancer within the past 5years, experiencing xerostomia and/or dysphagia. - Experiencing trismus or having removed part of the tongue during cancer treatment was also significantly associated with eating problems. - Problems with the ability to speak and express one-self were strongly associated with dysphonia, trismus, and dysphagia in multivariate analyses. - Having removed part of the tongue during cancer treatment was also significantly associated with reporting speech problems. |
| 85 | (Kosgallana et al., 2022) | Lip = 9.4%  Anterior two-thirds of the tongue = 31.8%  Buccal mucosa =29.4%  FOM= 10.6%  Hard palate =4.7% Lower and upper alveolar ridge = 2.4%  Retromolar trigone =11.8% | Sri Lanka | During treatment | OCA=85 | EORTC QLQ-OH - 15 | - | Modified Sinhala translated version of the EORTC QLQ-OH15 questionnaire was capable of detecting small changes due to treatments and a reliable valid tool to measure OHQoL of oral cancer patients who receive RT with or without chemotherapy. |
| 86 | (Kosgallana et al., 2023) | Lip = 2.2%  Anterior two-thirds of the tongue =40.0%  Buccal mucosa =22.2%  FOM= 13.3%  Hard palate =5.6% Lower and upper alveolar ridge = 1.1%  Retromolar trigone = 8.9%  More than two sites = 6.7% | Sri Lanka | During treatment | OCA=90 | EORTC QLQ-OH – 15 | - | - The median scores of eating problem domain peaked during last week of radiotherapy and reduced at three months post radiotherapy. - Gum and speech problem was higher during last week of radiotherapy than three months post radiotherapy. - The changes of OHQOL between the time frames were statistically significant. - Baseline OHQOL in relation to ‘gum and speech problem’ domain and ‘teeth’ item was identified as influential factors for OHQOL during the last week of radiotherapy. |
| 87 | (S. Garner et al., 2023) | Oral cavity= 54.05%  Oropharynx= 29.7%  Other = 10.8%  Unknown primary =5.4% | United Kingdom | Post treatment | HNC=37 | LORQv3 | - | - Functional problems relating to HNC treatment had a significant effect on OHQoL and persisted after rehabilitation. - Removable implant prostheses had more problems associated than fixed. - Regarding prostheses, more dissatisfaction was reported with lower compared to upper, - and for removable or combination versus fixed. |
| 88 | (L. Patton et al., 2023) | Oropharynx = 47.2%  Oral cavity = 15.4%  Salivary gland =9.6% Larynx/Hypopharynx =6.6%  Other =15.2% Unknown = 5.9% | USA | Post treatment | HNC=572 | EORTC QLQ -H&N35 | - | - The most persistently impacted OHQOL variables at 24 months included: dry mouth; sticky saliva, and senses problems. - Aspects of swallowing were most impacted by oropharyngeal tumor site, chemotherapy, and non-Hispanic ethnicity. - Problems with senses and dry mouth were worse with older age. - Dry mouth and sticky saliva increased more among men and those with oropharyngeal cancer, nodal involvement, and use of chemotherapy. - Problems with mouth opening were increased by chemotherapy and were more common among non-White and Hispanic individuals. - A 1000 cGy increase in RT dose was associated with a clinically meaningful change in difficulty swallowing solid food, dry mouth, sticky saliva, sense of taste, and senses problems. |
| 89 | (J Yang et al., 2023) | Nasopharynx = 100% | China | Post treatment | 96 | Geriatric Oral Health  Assessment Index (GOHAI) | - | - Statistically significant decrease in OHQoL after RT. - Functional limitation and pain and discomfort scores were significantly reduced, while those of psychological discomfort section were not significantly reduced. - The average dose received by the maxillary anterior teeth and oral cavity, tumor volume (GTVnx), and liking of the sweet food all affected GOHAI score. - When the average dose received by maxillary anterior teeth is greater than 28.78 Gy, there is a tendency in sweet food, the OHQoL will deteriorate. |
| 90 | (T Chiba et al., 2024) | Maxillary gingival cancer = 21.4%  Mandibular gingival cancer= 23.2%  Tongue =20.5%  FOM=10.7%  Pharyngeal=11.6%  Buccal mucosa=12.5% | Japan | Post treatment | OCA=112 | OHIP-14 | Food intake status, Gum gelly test | - Maxillary defect, tongue and soft palate tissue defect, and chemoradiotherapy were identified as factors that hindered the effectiveness of maxillofacial prosthetic treatment for improvement of the OHQoL. - On the other hand, reconstructive surgery was a factor that facilitated the improvement of OHQoL |
| 91 | (de Oliveira et al., 2024) | Lip=12.5%  Tongue=62.5%  Palate=25% | Brazil | During chemoradiation therapy | OCA=16 | OHIP-14 | Oral mucositis, DMFT | Comparing the daily and alternate-day photobiomodulation protocol, there were no statistically significant difference between the total and domain specific OHIP-14 scores in the two study arms |
| 92 | (C Herpel et al., 2023) | Nasal and paranasal sinus= 36.36%  Tongue or FOM = 27.27%  Maxilla and palate=22.7%  Mandible=4.5%  Tonsils=4.5%  Cheek=4.5% | Germany | During radiotherapy | Intervention group=9  Control group=13 | OHIP-14 | Jaw functional limitation scale | 3D-printed tissue retraction device was compared with conventional dental splints during RT, there were no statistically significant difference between the total and domain specific OHIP-14 scores in the two study groups |
| 93 | (Song et al., 2023) | Tongue =52.9% FOM= 19.1%  Soft palate = 8.9% Buccal mucosa = 7.8%  Others = 11.23% | China | Post treatment | DPAP group=57  PPMMF group = 32 | OHIP-14 | UW-QoL | Double-paddle peroneal artery perforator (DPAP) free flap significantly improved the patients’ OHQOL compared to pedicled pectoralis major myocutaneous flap (PPMMF) reconstruction |
| OHQoL-oral health related quality of life, HNC-head and neck cancer, RT-radiotherapy, WHO- world health organization, OCA-oral cancer, OP-oropharyngeal, FOM=floor of the mouth, OHIP-Oral Health Impact Profile, DMFT-decayed, missing filled teeth, EORTC QLQ–OH15-European Organization for Research and Treatment of Cancer Quality of Life Questionnaire Oral Health Module, EORTC QLQ-H&N -European Organization for Research and Treatment of Cancer Quality of Life Questionnaire Head and Neck Module, LORQv3 -Liverpool Oral Rehabilitation Questionnaire version 3, UW-QoL-University of Washington Quality of Life Scale, OIDP-Oral Impact on Daily Performance scale. SF- Sort form health survey | | | | | | | | |

Table S3: Studies on OPMD patients included in the systematic data synthesis (n=8)

|  | **Author-Date** | **Site** | **Country** | **Time of OHQoL assessment** | **Sample**  **Size** | **OHQoL**  **Tool** | **Other assessment** | **Main findings in relation to OHQoL** |
| --- | --- | --- | --- | --- | --- | --- | --- | --- |
| 1 | (Karbach et al., 2014) | Oral cavity | Germeny | Before treatment | Oral lichen planus (OLP)=73  Oral leukoplakia (OL)=44  Oral squamous cell carcinoma (OSCC)=37 | OHIP-G14 German version | - | - Patients with OL showed the lowest total OHIP-G 14 scores, patients with OLP, showed the highest scores, and patients with OSCC registered scores in between. - After comparing the dimensions, only ‘‘physical pain’’ and ‘‘social disability’’ differed significantly among the 3 groups. - Patients with OLP perceived more physical pain than patients with OSCC and OL showed the lowest impact for ‘‘physical pain’’ - For social disability,’’ the highest values were found in patients with OSCC followed by OLP and OL - No influence of age, smoking habits, and alcohol and OHQoL in the 3 groups - Women with OLP showed significantly higher scores on the OHIP than men with OLP. |
| 2 | (Alsoghier et al., 2021) | Tongue=41%  Buccal mucosa=27% Upper/lower gingiva=11%  Hard and soft palate=8% Lips and labial mucosa=6%  FOM=6% | United Kingdom | Before treatment | Oral lichen planus = 30  Oral leukoplakia=18  Chronic hyperplastic candidosis =4  Oral submucous fibrosis =2  Oral erythroplakia =1  Not recorded=27 | OHIP-14 | Hospital Anxiety and Depression Scale (HADS), Modified Dental Anxiety Scale (MDAS) | - A prevalence of 30% for anxiety, 16% for depression and 26% for emotional distress was reported. - Mean OHIP-14 score was 11.5 and physical pain and psychological disability were the most compromised domains. - With every additional comorbidity, there was a chance of increase on OHIP- 14 score - Oral dysplasia can adversely impact on the psychosocial well- being of affected persons. |
| 3 | (Gabrić, 2019) | Buccal mucosa=40.7% Tongue= 22.2%  Sublingual mucosa = 11.1%  Other=26% | Croatia | Post treatment | Leukoplakia= 27 | OHIP-14 | - | The mean OHIP total score was low indicating a better OHQoL with laser therapy, without any statistically significant differences between men and women in this group |
| 4 | (Matulić et al., 2019) | Buccal mucosa=33.3% | Croatia | Post treatmnt | Leukoplakia=54 | OHIP-14 | - | - Following Er:YAG and Er,Cr:YSGG lasers therapy, after 2 weeks from the initial ablation, the total OHIP-14 scores reduced significantly. - Both therapies were effective in improving the OHQoL. |
| 5 | (Memon et al., 2022) | Oral cavity | Pakistan | Post treatment | Oral submucous fibrosis =130 | OHIP-14 | - | The results showed that, apart from psychological disability, there was a significant improvement in all domains of OHQoL after treatment with intralesional injections of dexamethasone and hyaluronidase. |
| 6 | (Zucoloto et al., 2019) | Buccal mucosa=51.7%  Gingiva=32.1%  Tongue = 14.9%  Palate=1.1% | Brazil | Before treatment | Oral lichen planus=45  Oral lichenoid reactions=42  Controls=87 | OHIP-14 | Hamilton Anxiety Scale (HAM-A) | - Patients had higher scores for the OHIP-14 dimensions physiological discomfort and social limitation compared with controls. - In addition, higher scores for physical pain, physical disability, social disability, and handicap were detected among patients with greater disease severity, associated with anxiety |
| 7 | (Stolte et al., 2024) | Oral cavity | Germeny | Before treatment | Oral lichen planus=21  Controls = 17 | OHIP-14 | gene expression profile, antimicrobial peptides, oral disease severity score (ODSS) | - Patients had worse OHQoL compared to controls - Statistically significant differences in the domains, physical pain and psychological discomfort were reported. - OHQoL decreased with increasing ODSS score with a significant correlation. - Weak correlations were found between the expression of the anti-microbial peptides and the OHIP-14 score |
| 8 | (Warhekar et al., 2024) | Oral cavity | India | Before treatment | leukoplakia, erythroplakia, and oral submucous fibrosis = 500 | OHIP-14 | - | - Patients in the middle‑age group (e.g. 40‑60 years) reported the highest OHIP‑14 scores, indicating a greater negative impact on OHQoL - Significant impact was observed in the domains of functional limitations, physical pain, and psychological discomfort. - Female patients reported significantly higher OHIP‑14 scores than their male counterparts. |
| OPMD: oral potentially malignant disorders, OHIP: oral health impact profile | | | | | | | | |

Table S4: Quality assessment of included studies (n=101)

|  | **Reference** | **Checklist** | **Score** | **Grading** |
| --- | --- | --- | --- | --- |
| 1 | (Barrios et al., 2015a) | JBI-cross sectional | 8/8 | Good |
| 2 | (Barrios et al., 2015b) | JBI-cross sectional | 8/8 | Good |
| 3 | (Barrios et al., 2014) | JBI-cross sectional | 8/8 | Good |
| 4 | (de Melo et al., 2018) | JBI-cross sectional | 8/8 | Good |
| 5 | (de Melo et al., 2019) | JBI-cross sectional | 8/8 | Good |
| 6 | (Dholam et al., 2016) | JBI-cross sectional | 8/8 | Good |
| 7 | (Dholam et al., 2017) | JBI-cross sectional | 8/8 | Good |
| 8 | (Ettl, 2016) | JBI-cross sectional | 8/8 | Good |
| 9 | (Hagio et al., 2018) | JBI-cross sectional | 8/8 | Good |
| 10 | (Hassel et al., 2012) | JBI-cross sectional | 8/8 | Good |
| 11 | (Indrapriyadharshini et al., 2017) | JBI-cross sectional | 8/8 | Good |
| 12 | (Ishida et al., 2015) | JBI-RCT | 10/13 | Fair |
| 13 | (Kalaignan and Shree Mohan, 2018) | JBI-cross sectional | 5/8 | Fair |
| 14 | (Karbach et al., 2014) | JBI-cohort | 11/11 | Good |
| 15 | (Kumar et al., 2016) | JBI-RCT | 10/13 | Fair |
| 16 | (Li et al., 2017) | JBI-cross sectional | 8/8 | Good |
| 17 | (Li et al., 2013) | JBI-cross sectional | 8/8 | Good |
| 18 | (Li et al., 2016) | JBI-cohort | 9/11 | Good |
| 19 | (Linsen et al., 2009) | JBI-cross sectional | 8/8 | Good |
| 20 | (Maeda et al., 2018) | JBI-cohort | 10/11 | Good |
| 21 | (Malouf et al., 2003) | JBI-cohort | 10/11 | Good |
| 22 | (McMillan et al., 2004) | JBI-cross sectional | 8/8 | Good |
| 23 | (Mertens et al., 2016) | JBI-cross sectional | 8/8 | Good |
| 24 | (Pow et al., 2012) | JBI-cross sectional | 8/8 | Good |
| 25 | (Santos et al., 2017) | JBI-cohort | 10/11 | Good |
| 26 | (Schweyen et al., 2017) | JBI-cohort | 10/11 | Good |
| 27 | (Stuani et al., 2018) | JBI-cohort | 10/11 | Good |
| 28 | (Witsell et al., 2012) | JBI-RCT | 13/13 | Good |
| 29 | (Wolff, T J et al., 2004) | JBI-cross sectional | 8/8 | Good |
| 30 | (Yang et al., 2014) | JBI-cross sectional | 8/8 | Good |
| 31 | (Yuan et al., 2016) | JBI-cohort | 11/11 | Good |
| 32 | (Zhu et al., 2017) | JBI-cohort | 11/11 | Good |
| 33 | (Barkokebas et al., 2015) | JBI-cross sectional | 8/8 | Good |
| 34 | (Brandão et al., 2016) | JBI-RCT | 13/13 | Good |
| 35 | (Karayazgan-Saracoglu et al., 2017) | JBI-RCT | 10/13 | Fair |
| 36 | (Said et al., 2017) | JBI-cross sectional | 8/8 | Good |
| 37 | (Wang et al., 2019) | JBI-cross sectional | 8/8 | Good |
| 38 | (Yusa et al., 2017) | JBI-RCT | 10/13 | Fair |
| 39 | (Abed et al., 2023) | JBI-cross sectional | 8/8 | Good |
| 40 | (Anderson et al., 2021) | JBI-cross sectional | 8/8 | Good |
| 41 | (Aparna et al., 2022) | JBI-cross sectional | 8/8 | Good |
| 42 | (Binnal et al., 2021) | JBI-cross sectional | 8/8 | Good |
| 43 | (Čanković et al., 2022) | JBI-cross sectional | 8/8 | Good |
| 44 | (Coward et al., 2020) | JBI-cross sectional | 7/8 | Good |
| 45 | (Depeyre et al., 2020) | JBI-cross sectional | 8/8 | Good |
| 46 | (Dholam et al., 2020) | JBI-cohort | 6/11 | Fair |
| 47 | (Gondivkar et al., 2021) | JBI-cohort | 7/11 | Fair |
| 48 | (Gondivkar et al., 2021) | JBI-cross sectional | 8/8 | Good |
| 49 | (Huang et al., 2021) | JBI-cross sectional | 8/8 | Good |
| 50 | (Jung et al., 2019) | JBI-cross sectional | 7/8 | Good |
| 51 | (Maqbool et al., 2021) | JBI-case control | 10/10 | Good |
| 52 | (Martins et al., 2021) | JBI-RCT | 12/13 | Good |
| 53 | (Naidu et al., 2019) | JBI-cross sectional | 8/8 | Good |
| 54 | (Nascimento et al., 2019) | JBI-cross sectional | 7/8 | Good |
| 55 | (Pereira et al., 2020) | JBI-RCT | 11/13 | Good |
| 56 | (Pieralli et al., 2021) | JBI-RCT | 5/13 | Poor |
| 57 | (Soldera et al., 2020) | JBI-cross sectional | 8/8 | Good |
| 58 | (Stefano et al., 2019) | JBI-cohort | 5/11 | Poor |
| 59 | (Tesic et al., 2020) | JBI-cross sectional | 8/8 | Good |
| 60 | (Westgaard et al., 2021) | JBI-cross sectional | 8/8 | Good |
| 61 | (Winter et al., 2021) | JBI-cohort | 9/11 | Good |
| 62 | (Xu et al., 2022) | JBI-cross sectional | 8/8 | Good |
| 63 | (Yanamoto et al., 2020) | JBI-RCT | 6/13 | Poor |
| 64 | (Zhang et al., 2020) | JBI-cross sectional | 6/8 | Good |
| 65 | (Zhu et al., 2017) | JBI-cohort | 9/11 | Good |
| 66 | (Fromm et al., 2019) | JBI-cross sectional | 8/8 | Good |
| 67 | (Xiao et al., 2019) | JBI-cross sectional | 8/8 | Good |
| 68 | (Chouksey et al., 2023) | JBI-case control | 5/10 | Fair |
| 69 | (P Jehn et al., 2024) | JBI-cross sectional | 6/8 | Good |
| 70 | (Shirakawa et al., 2024) | JBI-cross sectional | 6/8 | Good |
| 71 | (Ghorbani et al., 2023) | JBI-case control | 7/10 | Fair |
| 72 | (Martins et al., 2021) | JBI-RCT | 11/13 | Good |
| 73 | (Song et al., 2023) | JBI-case control | 8/10 | Good |
| 74 | (Caminha et al., 2024) | JBI-cross sectional | 6/8 | Good |
| 75 | (Winter et al., 2023) | JBI-cross sectional | 8/8 | Good |
| 76 | (Aguiar et al., 2024) | JBI-cross sectional | 8/8 | Good |
| 77 | (Afzal et al., n.d.) | JBI-cross sectional | 4/8 | Fair |
| 78 | (Zeman-Kuhnert et al., 2024) | JBI-cross sectional | 6/8 | Good |
| 79 | (Jiang et al., 2024) | JBI-RCT | 8/13 | Fair |
| 80 | (Al-Aroomi et al., 2024) | JBI-case control | 8/10 | Good |
| 81 | (Qayyum et al., 2023) | JBI-cross sectional | 5/8 | Fair |
| 82 | (Inês Rodrigues et al., 2023) | JBI-cross sectional | 6/8 | Good |
| 83 | (Neelanjali Matapathi et al., 2022) | JBI-cross sectional | 4/8 | Fair |
| 84 | (Reichal and Prethipa, 2024) | JBI-cross sectional | 4/8 | Fair |
| 85 | (Qamar et al., 2024) | JBI-cross sectional | 6/8 | Good |
| 86 | (Andreassen and Hadler‐Olsen, 2023) | JBI-cross sectional | 5/8 | Fair |
| 87 | (Kosgallana et al., 2022) | JBI-cross sectional | 6/8 | Good |
| 88 | (Kosgallana et al., 2023) | JBI-cross sectional | 6/8 | Good |
| 89 | (S. J. Garner et al., 2023) | JBI-cross sectional | 7/8 | Good |
| 90 | (L. L. Patton et al., 2023) | JBI-cohort | 7/11 | Fair |
| 91 | (Jing Yang et al., 2023) | JBI-cross sectional | 6/8 | Good |
| 92 | (Takahiro Chiba et al., 2024) | JBI-cohort | 6/11 | Fair |
| 93 | (Oliveira et al., 2024) | JBI-RCT | 11/13 | Good |
| 94 | (Christopher Herpel et al., 2023) | JBI-RCT | 8/13 | Fair |
| 95 | (Alsoghier et al., 2021) | JBI-cross sectional | 6/8 | Good |
| 96 | (Gabrić, 2019) | JBI-cross sectional | 3/8 | Poor |
| 97 | (Matulić et al., 2019) | JBI-cross sectional | 5/8 | Fair |
| 98 | (Memon et al., 2022) | JBI-cross sectional | 6/8 | Good |
| 99 | (Zucoloto et al., 2019) | JBI-cross sectional | 5/8 | Fair |
| 100 | (Stolte et al., 2024) | JBI-case control | 5/10 | Fair |
| 101 | (Warhekar et al., 2024) | JBI-cross sectional | 3/8 | Poor |
| JBI-Joanna Briggs Institute, RCT-randomized control trial, scores are presented as the statements marked as ‘Yes’ out of the total number of statements, Gradings for each tool was decided as follows. JBI-cross sectional tool 8-6 good, 5-3 fair and <3 poor, JBI-cohort tool 9-11 good, 6-8 fair and <6 poor, JBI-RCT tool 11-13 good, 8-10 fair and <8 poor. JBI-case control tool 10-8 good, 7-5 fair and less than 5 poor. | | | | |

**References**

Abed, H., Reilly, D., Burke, M., Sharka, R., Daly, B., 2023. The association between dental arch length and oral health‐related quality of life in head and neck cancer patients post‐radiotherapy. Special Care in Dentistry 43, 111–118. https://doi.org/10.1111/scd.12755

Afzal, A., Alam, J., Saddique, A., Ahmed, N., n.d. Assessment of Common Oral Dysfunction and Their Impact on Oral Health Related Quality of Life in Post Treatment Patients of Oral Squamous Cell Carcinoma : A Prospective Study.

Aguiar, A.W.P.B.D., Lins, L.A.N., Fonte, A.L.F.D., Albuquerque, R.F.D., Leão, J.C., Silva, I.H.M., 2024. 3D radiotherapy in the parotid gland and its dosimetric relationship with salivary flow and quality of life in patients with head and neck cancer. Braz. J. Oral Sci. 23, e246798. https://doi.org/10.20396/bjos.v23i00.8666798

Al-Aroomi, M., Al-Worafi, N., Ma, Y., Alkebsi, K., Mohamed, A., Jiang, C., 2024. Patient-reported outcomes after oral cancer reconstructions with radial and ulnar forearm-free flaps. ORAL DISEASES. https://doi.org/10.1111/odi.14968

Alsoghier, A., Riordain, R.N., Fedele, S., Porter, S., 2021. Psychosocial impacts of oral epithelial dysplasia. Journal of Oral Pathology and Medicine 50, 700–707. https://doi.org/10.1111/jop.13173

Anderson, G., Ebadi, M., Vo, K., Novak, J., Govindarajan, A., Amini, A., 2021. An updated review on head and neck cancer treatment with radiation therapy. Cancers 13, 1–12. https://doi.org/10.3390/cancers13194912

Andreassen, R., Hadler‐Olsen, E., 2023. Eating and speech problems in oral and pharyngeal cancer survivors – Associations with treatment‐related side‐effects and time since diagnosis. Special Care in Dentistry 43, 561–571. https://doi.org/10.1111/scd.12791

Andreassen, R., Hadler-Oslen, E., 2022. Eating and speech problems in oral and pharyngeal cancer survivors - Associations with treatment-related side-effects and time since diagnosis. SPECIAL CARE IN DENTISTRY. https://doi.org/10.1111/scd.12791

Andreassen, R., Jönsson, B., Hadler-Olsen, E., 2022. Oral health related quality of life in long-term survivors of head and neck cancer compared to a general population from the seventh Tromsø study. BMC Oral Health 22, 100. https://doi.org/10.1186/s12903-022-02140-2

Aparna, K.S., Manjunath, P.P., Sowmya, K.R., 2022. Oral Health Status and Quality of Life Among Head and Neck Cancer Subjects Receiving Radiotherapy. Journal of International Dental and Medical Research 15, 263–267.

Barkokebas, A., Silva, I.H.M., De Andrade, S.C., Carvalho, A.A.T., Gueiros, L.A.M., Paiva, S.M., Leão, J.C., 2015. Impact of oral mucositis on oral‐health‐related quality of life of patients diagnosed with cancer. J Oral Pathology Medicine 44, 746–751. https://doi.org/10.1111/jop.12282

Barrios, R., Bravo, M., Gil-Montoya, J.A., Martínez-Lara, I., García-Medina, B., Tsakos, G., 2015a. Oral and general health-related quality of life in patients treated for oral cancer compared to control group. Health and Quality of Life Outcomes 13, 1–8. https://doi.org/10.1186/s12955-014-0201-5

Barrios, R., Tsakos, G., García-Medina, B., Martínez-Lara, I., Bravo, M., 2014. Oral health-related quality of life and malnutrition in patients treated for oral cancer. Supportive Care in Cancer 22, 2927–2933. https://doi.org/10.1007/s00520-014-2281-5

Barrios, R., Tsakos, G., Gil-Montoya, J.A., Montero, J., Bravo, M., 2015b. Association between general and oral health-related quality of life in patients treated for oral cancer. Medicina Oral, Patologia Oral y Cirugia Bucal 20, e678–e684. https://doi.org/10.4317/medoral.20714

Binnal, A., Rajesh, G., Prakash Saxena, P.U., Banerjee, S., Denny, C., Tadakamadla, S.K., 2021. Health-related quality of life among oral and oropharyngeal cancer patients: An exploratory study. Oral diseases. https://doi.org/10.1111/odi.13772

Brandão, T.B., Vechiato Filho, A.J., Prado Ribeiro, A.C., Gebrim, E.M.M.S., Bodard, A.-G., Da Silva, D.P., Santos-Silva, A.R., Ishida, L.C., Dias, R.B., 2016. Evaluation of use of acrylic resin-based surgical guide in the function and quality of life provided by mandibular prostheses with microvascular free fibula flap: A four-year, randomized, controlled trial. The Journal of Prosthetic Dentistry 116, 457-463.e2. https://doi.org/10.1016/j.prosdent.2016.02.012

Caminha, R.D.G., Caldas, R.J., Bueno, I.C.M., Scaraficci, A.C., Da Silva Santos, P.S., 2024. A time frame evaluation of the oral health-related quality of life among patients with head and neck cancer. Oral Oncology Reports 11, 100593. https://doi.org/10.1016/j.oor.2024.100593

Čanković, M., Tešić, M., Jevtić, M., Stevanović, D., Jovanović, Mb., Kostić, D., Antić, J., Trivić, Sk., 2022. Predictors of health-related quality of life in Serbian patients with head and neck cancer. Med Oral e340–e350. https://doi.org/10.4317/medoral.25274

Chiba, T, Izumita, K., Koyama, S., Sato, N., Tagaino, R., Hatakeyama, T., Sasaki, K., 2024. Effects of maxillofacial prosthetic treatment on oral health-related quality of life and masticatory ability of patients with head and neck tumors. JOURNAL OF ORAL SCIENCE 66, 30–36. https://doi.org/10.2334/josnusd.23-0162

Chiba, Takahiro, Izumita, K., Koyama, S., Sato, N., Tagaino, R., Hatakeyama, T., Sasaki, K., 2024. Effects of maxillofacial prosthetic treatment on oral health-related quality of life and masticatory ability of patients with head and neck tumors. J Oral Sci 66, 30–36. https://doi.org/10.2334/josnusd.23-0162

Chouksey, G., Dugad, J., Gupta, V., Dholam, K., Goel, P., Choure, R., 2023. Oral health-related quality of life after rehabilitation with maxillary obturators: A comparison of patients with maxillectomy defects associated with cancer and post-COVID-19 mucormycosis. The Journal of Prosthetic Dentistry S0022391323007576. https://doi.org/10.1016/j.prosdent.2023.11.009

Coward, T.J., Richards, R., Fenlon, M.R., Scott, B.J.J., 2020. Effect of obturators on facial form following surgery for head and neck cancer and impact on the perception of appearance. Journal of Dentistry 92, 103230. https://doi.org/10.1016/j.jdent.2019.103230

de Melo, N.B., Bernardino, Í. de M., de Melo, D.P., Gomes, D.Q.C., Bento, P.M., 2018. Head and neck cancer, quality of life, and determinant factors: a novel approach using decision tree analysis. Oral surgery, oral medicine, oral pathology and oral radiology 126, 486–493. https://doi.org/10.1016/j.oooo.2018.07.055

de Melo, N.B., de Sousa, V.M., Bernardino, M.M., de Melo, D.P., Gomes, D.Q.C., Bento, P.M., 2019. Oral health related quality of life and determinant factors in patients with head and neck cancer. Medicina oral, patologia oral y cirugia bucal 24, e281–e289. https://doi.org/10.4317/medoral.22670

de Oliveira, F., Borges, M., Malta, C., de Moura, J., Forte, C., Barbosa, J., Silva, P., Dantas, T., 2024. Comparison of a daily and alternate-day photobiomodulation protocol in the prevention of oral mucositis in patients undergoing radiochemotherapy for oral cancer: a triple-blind, controlled clinical trial. MEDICINA ORAL PATOLOGIA ORAL Y CIRUGIA BUCAL 29, e430–e440. https://doi.org/10.4317/medoral.26436

Depeyre, A., Pereira, B., Pham-Dang, N., Barthélémy, I., Hennequin, M., 2020. Impairments in Food Oral Processing in Patients Treated for Tongue Cancer. Dysphagia 35, 494–502. https://doi.org/10.1007/s00455-019-10054-5

Dholam, K., Chouksey, G., Dugad, J., 2020. Impact of Oral Rehabilitation on Patients with Head and Neck Cancer: Study of 100 Patients with Liverpool Oral Rehabilitation Questionnaire and the Oral Health Impact Profile. Indian J Otolaryngol Head Neck Surg 72, 308–312. https://doi.org/10.1007/s12070-020-01801-4

Dholam, K., Chouksey, G., Dugad, J., 2016. Oral health-related quality of life after prosthetic rehabilitation in patients with oral cancer: A longitudinal study with the Liverpool Oral Rehabilitation Questionnaire version 3 and Oral Health Impact Profile-14 questionnaire. Indian J Cancer 53, 256. https://doi.org/10.4103/0019-509X.197716

Dholam, K.P., Dugad, J.A., Sadashiva, K.M., 2017. Impact of oral rehabilitation on patients with head and neck cancer: A study using the Liverpool Oral Rehabilitation Questionnaire and the Oral Health Impact Profile-14. The Journal of Prosthetic Dentistry 117, 559–562. https://doi.org/10.1016/j.prosdent.2016.06.019

Ettl, T., 2016. Impact of radiotherapy on implant-based prosthetic rehabilitation in patients with head and neck cancer: A prospective observational study on implant survival and quality of life-Preliminary results. Facial Surgery.

Fromm, L., Gotfredsen, K., Wessel, I., Øzhayat, E.B., 2019. Oral health‐related quality of life, oral aesthetics and oral function in head and neck cancer patients after oral rehabilitation. J of Oral Rehabilitation 46, 738–746. https://doi.org/10.1111/joor.12806

Gabrić, D., 2019. Evaluation of Innovative Digitally Controlled Er:YAG Laser in Surgical Treatment of Oral Leukoplakia – a Preliminary Study. ACC 58. https://doi.org/10.20471/acc.2019.58.04.07

Garner, S., Patel, S., Pollard, A., Jerreat, M., 2023. Post-treatment evaluation of oral health-related quality of life in head and neck cancer patients after dental implant rehabilitation. BRITISH DENTAL JOURNAL. https://doi.org/10.1038/s41415-023-5460-2

Garner, S.J., Patel, S., Pollard, A.J., Jerreat, M.P., 2023. Post-treatment evaluation of oral health-related quality of life in head and neck cancer patients after dental implant rehabilitation. Br Dent J. https://doi.org/10.1038/s41415-023-5460-2

Ghorbani, Z., Manifar, S., Bohloli, G., Aghakouchakzadeh, A., Mirzaei, A., 2023. Oral health-related quality of life in patients with oral squamous cell carcinoma: A case–control study. Dental Research Journal 20. https://doi.org/10.4103/1735-3327.372653

Gondivkar, S., Gadbail, A., Sarode, S., Dasgupta, S., Sharma, B., Hedaoo, A., Sharma, A., Sarode, G., Yuwanati, M., Gondivkar, R., Patil, S., Gaikwad, R., 2021. Prevalence of Trismus and Its Impact on Oral Health-Related Quality of Life in Patients Treated for Oral Squamous Cell Carcinoma. Asian Pac J Cancer Prev 22, 2437–2444. https://doi.org/10.31557/APJCP.2021.22.8.2437

Gondivkar, S.M., Gadbail, A.R., Sarode, S.C., Hedaoo, A., Dasgupta, S., Sharma, B., Sharma, A., Gondivkar, R.S., Yuwanati, M., Patil, S., Gaikwad, R.N., 2021. Oral and general health-related quality of life in oral squamous cell carcinoma patients- comparative analysis of different treatment regims. Journal of Oral Biology and Craniofacial Research 11, 125–131. https://doi.org/10.1016/j.jobcr.2021.01.004

Hagio, M., Ishizaki, K., Ryu, M., Nomura, T., Takano, N., Sakurai, K., 2018. Maxillofacial prosthetic treatment factors affecting oral health-related quality of life after surgery for patients with oral cancer. The Journal of Prosthetic Dentistry 119, 663–670. https://doi.org/10.1016/j.prosdent.2017.05.017

Hassel, A.J., Danner, D., Freier, K., Hofele, C., Becker-Bikowski, K., Engel, M., 2012. Oral health-related quality of life and depression/anxiety in long-term recurrence-free patients after treatment for advanced oral squamous cell cancer. Journal of Cranio-Maxillofacial Surgery 40, e99–e102. https://doi.org/10.1016/j.jcms.2011.05.011

Herpel, C, Held, T., Labis, C., Christ, L., Lang, K., Regnery, S., Eichkorn, T., Lentz-Hommertgen, A., Jaekel, C., Moratin, J., Semmelmayer, K., Moutsis, T., Plath, K., Ristow, O., Freudlsperger, C., Adeberg, S., Debus, J., Rammelsberg, P., Schwindling, F., 2023. Oral Sequelae after Head and Neck Radiotherapy: RCT Comparing 3D-Printed Tissue Retraction Devices with Conventional Dental Splints. JOURNAL OF CLINICAL MEDICINE 12. https://doi.org/10.3390/jcm12082789

Herpel, Christopher, Held, T., Labis, C., Christ, L., Lang, K., Regnery, S., Eichkorn, T., Lentz-Hommertgen, A., Jaekel, C., Moratin, J., Semmelmayer, K., Moutsis, T.T., Plath, K., Ristow, O., Freudlsperger, C., Adeberg, S., Debus, J., Rammelsberg, P., Schwindling, F.S., 2023. Oral Sequelae after Head and Neck Radiotherapy: RCT Comparing 3D-Printed Tissue Retraction Devices with Conventional Dental Splints. JCM 12, 2789. https://doi.org/10.3390/jcm12082789

Huang, B.-S., Chung, C.-F., Chang, Y.-L., Lee, L.-Y., Peng, H.-L., Chen, S.-C., 2021. Body mass index and self-care behaviors related to oral health–related quality of life in patients with oral squamous cell carcinoma within three months posttreatment. Support Care Cancer 29, 2239–2248. https://doi.org/10.1007/s00520-020-05737-x

Indrapriyadharshini, K., Madankumar, P., Karthikeyan, G., 2017. Oral health-related quality of life in patients treated for oral malignancy at Kanchipuram district, India: A cross-sectional study. Indian J Cancer 54, 11. https://doi.org/10.4103/ijc.IJC_116_17

Ishida, S., Shibuya, Y., Kobayashi, M., Komori, T., 2015. Assessing stomatognathic performance after mandibulectomy according to the method of mandibular reconstruction. International Journal of Oral and Maxillofacial Surgery 44, 948–955. https://doi.org/10.1016/j.ijom.2015.03.011

Jehn, P, Korn, P., Spalthoff, S., Schiller, M., Lentge, F., Bolstorff, I., Tavassol, F., Gellrich, N., Rahlf, B., 2024. Dental rehabilitation in irradiated oral cancer patients using patient-specific dental implants - Clinical outcome and oral health-related quality of life. JOURNAL OF STOMATOLOGY ORAL AND MAXILLOFACIAL SURGERY 125. https://doi.org/10.1016/j.jormas.2023.101674

Jehn, Philipp, Korn, P., Spalthoff, S., Schiller, M., Lentge, F., Bolstorff, I., Tavassol, F., Gellrich, N.-C., Rahlf, B., 2024. Dental rehabilitation in irradiated oral cancer patients using patient-specific dental implants – Clinical outcome and oral health-related quality of life. Journal of Stomatology, Oral and Maxillofacial Surgery 125, 101674. https://doi.org/10.1016/j.jormas.2023.101674

Jiang, N., Zhao, Y., M�rtensson, J., Stensson, M., 2024. The effects of an integrated supportive programme on oral health in patients with head and neck cancer undergoing radiotherapy: A randomized controlled trial. INTERNATIONAL JOURNAL OF DENTAL HYGIENE. https://doi.org/10.1111/idh.12801

Jung, Y.-S., Park, E.-Y., Sohn, H.-O., 2019. Oral Health Status and Oral Health-related Quality of Life According to Presence or Absence of Mucositis in Head and Neck Cancer Patients. J Cancer Prev 24, 43–47. https://doi.org/10.15430/JCP.2019.24.1.43

Kalaignan, P., Shree Mohan, J., 2018. Oral Health Related Quality Of Life with Mandibular Resection Prosthesis. Biomed. Pharmacol. J. 11, 1423–1428. https://doi.org/10.13005/bpj/1506

Karayazgan-Saracoglu, B., Atay, A., Korkmaz, C., Gunay, Y., 2017. Quality of life assessment of implant-retained overdentures and fixed metal-acrylic resin prostheses in patients with marginal mandibulectomy. The Journal of Prosthetic Dentistry 118, 551–560. https://doi.org/10.1016/j.prosdent.2017.01.025

Karbach, J., Al-Nawas, B., Moergel, M., Daubländer, M., 2014. Oral Health-Related Quality of Life of Patients With Oral Lichen Planus, Oral Leukoplakia, or Oral Squamous Cell Carcinoma. Journal of Oral and Maxillofacial Surgery 72, 1517–1522. https://doi.org/10.1016/J.JOMS.2014.04.008

Kosgallana, S., Jayasekara, P., Abeysinghe, P., Hjermstad, M., Lalloo, R., 2022. Translation and validation of Sinhala version of modified EORTC QLQ-OH15 in oral cancer patients who receive radiotherapy with or without chemotherapy in Sri Lanka. BMC ORAL HEALTH 22. https://doi.org/10.1186/s12903-022-02392-y

Kosgallana, S., Jayasekara, P., Abeysinghe, P., Lalloo, R., 2023. Oral health related quality of life of oral cancer patients treated with radiotherapy alone or with chemotherapy in a tertiary referral centre in Sri Lanka. BMC Oral Health 23, 162. https://doi.org/10.1186/s12903-023-02854-x

Kumar, V.V., Jacob, P.C., Ebenezer, S., Kuriakose, M.A., Kekatpure, V., Baliarsing, A.S., Al-Nawas, B., Wagner, W., 2016. Implant supported dental rehabilitation following segmental mandibular reconstruction- quality of life outcomes of a prospective randomized trial. Journal of Cranio-Maxillofacial Surgery 44, 800–810. https://doi.org/10.1016/j.jcms.2016.04.013

Li, N., Otomaru, T., Taniguchi, H., 2017. Sleep quality in long-term survivors of head and neck cancer: preliminary findings. Support Care Cancer 25, 3741–3748. https://doi.org/10.1007/s00520-017-3804-7

Li, W., Yang, Y., Xu, Z., Liu, F., Cheng, Y., Xu, L., Sun, C., 2013. Assessment of quality of life of patients with oral cavity cancer who have had defects reconstructed with free anterolateral thigh perforator flaps. British Journal of Oral and Maxillofacial Surgery 51, 497–501. https://doi.org/10.1016/j.bjoms.2012.09.005

Li, W., Zhang, P., Li, R., Liu, Y., Kan, Q., 2016. Radial free forearm flap versus pectoralis major pedicled flap for reconstruction in patients with tongue cancer: Assessment of quality of lif. Med Oral 0–0. https://doi.org/10.4317/medoral.21274

Linsen, S., Schmidt-Beer, U., Fimmers, R., Grüner, M., Koeck, B., 2009. Craniomandibular Pain, Bite Force, and Oral Health-Related Quality of Life in Patients with Jaw Resection. Journal of Pain and Symptom Management 37, 94–106. https://doi.org/10.1016/j.jpainsymman.2006.12.019

Maeda, M., Hirose, M., Wada, K., Kishimoto, M., Akashi, M., Kimoto, A., Komori, T., Shibuya, Y., 2018. Elucidating the masticatory function and oral quality of life according to the range of mandibulectomy. Journal of Oral and Maxillofacial Surgery, Medicine, and Pathology 30, 220–224. https://doi.org/10.1016/j.ajoms.2018.01.004

Malouf, J.G., Aragon, C., Henson, B.S., Eisbruch, A., Ship, J.A., 2003. Influence of parotid-sparing radiotherapy on xerostomia in head and neck cancer patients. Cancer Detection and Prevention 27, 305–310. https://doi.org/10.1016/S0361-090X(03)00095-3

Maqbool, S., Siddique, S., Mehak, A., Ali, Q., Asghar, S.K., Shahid, F., 2021. Evaluation of Oral Health Related Quality of Life in Patients Diagnosed with Head and Neck Cancer Receiving Radiotherapy. PJMHS 15, 3119–3122. https://doi.org/10.53350/pjmhs2115113119

Martins, A.F.L., Morais, M.O., De Sousa-Neto, S.S., De Jesus, A.P.G., Nogueira, T.E., Valadares, M.C., Freitas, N.M.A., Batista, A.C., Leles, C.R., Mendonça, E.F., 2021. Photobiomodulation reduces the impact of radiotherapy on oral health-related quality of life due to mucositis-related symptoms in head and neck cancer patients. Lasers Med Sci 36, 903–912. https://doi.org/10.1007/s10103-020-03167-z

Matapathi, N, Shenoy, V., Shenoy, R., Miranda, G., Upadhya, M., Mehendale, A., Bangera, B., Shenoy, K., 2022. Evaluation of the quality of life of patients with maxillofacial defects after prosthodontic rehabilitation: A cross-sectional study. JOURNAL OF CANCER RESEARCH AND THERAPEUTICS 18, S219–S225. https://doi.org/10.4103/jcrt.JCRT_889_20

Matapathi, Neelanjali, Shenoy, V.K., Shenoy, R., Miranda, G.A., Upadhya, M., Mehendale, A., Bangera, B., Shenoy, K.K., 2022. Evaluation of the quality of life of patients with maxillofacial defects after prosthodontic rehabilitation: A cross-sectional study. Journal of Cancer Research and Therapeutics 18, S219–S225. https://doi.org/10.4103/jcrt.JCRT_889_20

Matulić, N., Bago, I., Sušić, M., Gjorgievska, E., Kotarac Knežević, A., Gabrić, D., 2019. Comparison of Er:YAG and Er,Cr:YSGG Laser in the Treatment of Oral Leukoplakia Lesions Refractory to the Local Retinoid Therapy. Photobiomodulation, Photomedicine, and Laser Surgery 37, 362–368. https://doi.org/10.1089/photob.2018.4560

McMillan, A.S., Pow, E.H.N., Leung, W.K., Wong, M.C.M., Kwong, D.L.W., 2004. Oral health‐related quality of life in southern Chinese following radiotherapy for nasopharyngeal carcinoma. J of Oral Rehabilitation 31, 600–608. https://doi.org/10.1111/j.1365-2842.2004.01383.x

Memon, A.B., Rahman, A.A.U., Channar, K.A., Zafar, M.S., Kumar, N., 2022. Evaluating the Oral-Health-Related Quality of Life of Oral Submucous Fibrosis Patients before and after Treatment Using the OHIP-14 Tool. IJERPH 19, 1821. https://doi.org/10.3390/ijerph19031821

Mertens, C., De San Jose Gonzalez, J., Freudlsperger, C., Bodem, J., Krisam, J., Hoffmann, J., Engel, M., 2016. Implant-prosthetic rehabilitation of hemimaxillectomy defects with CAD/CAM suprastructures. Journal of Cranio-Maxillofacial Surgery 44, 1812–1818. https://doi.org/10.1016/j.jcms.2016.08.009

Naidu, G., Shukla, S., Nagi, R., Jain, S., Makkad, R., 2019. Evaluation of oral health related quality of life in subjects diagnosed with head and neck malignancies undergoing chemotherapy, radiotherapy, and surgery. J Indian Acad Oral Med Radiol 31, 228. https://doi.org/10.4103/jiaomr.jiaomr_71_19

Nascimento, M., Farias, A., Carvalho, A., Albuquerque, R., Ribeiro, L., Leao, J., Silva, I., 2019. Impact of xerostomia on the quality of life of patients submitted to head and neck radiotherapy. Med Oral 0–0. https://doi.org/10.4317/medoral.23131

Oliveira, Fm., Borges, Mm., Malta, Ce., Moura, Jf., Forte, Cp., Barbosa, Jv., Silva, Pg., Dantas, Ts., 2024. Comparison of a daily and alternate-day photobiomodulation protocol in the prevention of oral mucositis in patients undergoing radiochemotherapy for oral cancer: a triple-blind, controlled clinical trial. Med Oral e430–e440. https://doi.org/10.4317/medoral.26436

Patton, L., Helgeson, E., Brennan, M., Treister, N., Sollecito, T., Schmidt, B., Lin, A., Chera, B., Lalla, R., 2023. Oral health-related quality of life after radiation therapy for head and neck cancer: the OraRad study. SUPPORTIVE CARE IN CANCER 31. https://doi.org/10.1007/s00520-023-07750-2

Patton, L.L., Helgeson, E.S., Brennan, M.T., Treister, N.S., Sollecito, T.P., Schmidt, B.L., Lin, A., Chera, B.S., Lalla, R.V., 2023. Oral health-related quality of life after radiation therapy for head and neck cancer: the OraRad study. Support Care Cancer 31, 286. https://doi.org/10.1007/s00520-023-07750-2

Pereira, N.F., López, R.M., Toporcov, T.N., Schmerling, C.K., Cicco, R.D., Michel-Crosato, E., Biazevic, M.G.H., 2020. Association between oral hygiene and head and neck cancer in Brazil. Revista brasileira de epidemiologia = Brazilian journal of epidemiology 23, e200094–e200094. https://doi.org/10.1590/1980-549720200094

Pereira, R.M.D.S., Bastos, M.D.R., Ferreira, M.P., De Freitas, O., De Macedo, L.D., De Oliveira, H.F., Ricz, H.M.A., Motta, A.C.F., Macedo, A.P., Tirapelli, C., Pedrazzi, V., 2020. Topical pilocarpine for xerostomia in patients with head and neck cancer treated with radiotherapy. Oral Diseases 26, 1209–1218. https://doi.org/10.1111/odi.13343

Pieralli, S., Spies, B.C., Schweppe, F., Preissner, S., Nelson, K., Heiland, M., Nahles, S., 2021. Retrospective long‐term clinical evaluation of implant‐prosthetic rehabilitations after head and neck cancer therapy. Clinical Oral Implants Res 32, 470–486. https://doi.org/10.1111/clr.13716

Pow, E.H.N., Kwong, D.L.W., Sham, J.S.T., Lee, V.H.F., Ng, S.C.Y., 2012. Can Intensity-Modulated Radiotherapy Preserve Oral Health-Related Quality of Life of Nasopharyngeal Carcinoma Patients? International Journal of Radiation Oncology*Biology*Physics 83, e213–e221. https://doi.org/10.1016/j.ijrobp.2011.12.040

Qamar, S., Rozi, S., Sawani, S., Awan, M.S., Akhtar, S., Siddiqui, M.I., Abbas, S.A., Taimoor, S., Raza Khan, F., 2024. Oral health related quality of life in head and neck cancer survivors within the first year following treatment: a cross-sectional study in Karachi, Pakistan. Sci Rep 14, 2560. https://doi.org/10.1038/s41598-024-52813-x

Qayyum, Z., Khan, Z.A., Maqsood, A., Prabhu, N., Saad Alqarni, M., Bader, A.K., Issrani, R., Abbasi, M.S., Ahmed, N., Sghaireen, M.G., Heboyan, A., 2023. Outcome Assessment after Reconstruction of Tumor-Related Mandibular Defects Using Free Vascularized Fibular Flap—A Clinical Study. Healthcare 11, 193. https://doi.org/10.3390/healthcare11020193

Reichal, P., Prethipa, R., 2024. A Comprehensive Retrospective Institutional Study for Decoding Oral Squamous Cell Carcinoma. CUREUS JOURNAL OF MEDICAL SCIENCE 16. https://doi.org/10.7759/cureus.54001

Rodrigues, Inês, Botelho, J., Machado, V., Proença, L., Mendes, J.J., Zagalo, C., 2023. Profiling oral health status, values, and related quality of life in patients with oral cancer: a pilot study. Front. Oral. Health 4, 1268657. https://doi.org/10.3389/froh.2023.1268657

Rodrigues, I, Botelho, J., Machado, V., Proen�a, L., Mendes, J., Zagalo, C., 2023. Profiling oral health status, values, and related quality of life in patients with oral cancer: a pilot study. FRONTIERS IN ORAL HEALTH 4. https://doi.org/10.3389/froh.2023.1268657

Said, M.M., Otomaru, T., Yeerken, Y., Taniguchi, H., 2017. Masticatory function and oral health-related quality of life in patients after partial maxillectomies with closed or open defects. The Journal of Prosthetic Dentistry 118, 108–112. https://doi.org/10.1016/j.prosdent.2016.11.003

Santos, P.S.S., Cremonesi, A.L., Quispe, R.A., Rubira, C.M.F., 2017. The impact of oral health on quality of life in individuals with head and neck cancer after radiotherapy: the importance of dentistry in psychosocial issues. Acta Odontol. Latinoam. 30.

Schweyen, R., Kuhnt, T., Wienke, A., Eckert, A., Hey, J., 2017. The impact of oral rehabilitation on oral health-related quality of life in patients receiving radiotherapy for the treatment of head and neck cancer. Clin Oral Invest 21, 1123–1130. https://doi.org/10.1007/s00784-016-1874-4

Shirakawa, J., Kaneuji, T., Matsuno, D., Nagata, J., Hirayama, B., Tanaka, F., Nakamura, Y., Yamashita, Y., 2024. Correlation during the extent of surgical resection, oral function and quality of life after tongue cancer surgery: Single-institution study. Journal of Stomatology, Oral and Maxillofacial Surgery 125, 101907. https://doi.org/10.1016/j.jormas.2024.101907

Soldera, E.B., Ortigara, G.B., Bonzanini, L.I.L., Schulz, R.E., Danesi, C.C., Antoniazzi, R.P., Linhares Ferrazzo, K., 2020. Clinical and sociodemographic factors associated with oral health‐related quality of life in survivors of head and neck cancer. Head & Neck 42, 886–897. https://doi.org/10.1002/hed.26063

Song, P., Li, J., Yang, D., Hu, K., Zhao, T., 2023. Assessment of quality of life after soft tissue resection of head and neck carcinoma and reconstruction with double-paddle peroneal artery perforator free flap. British Journal of Oral and Maxillofacial Surgery 61, 176–180. https://doi.org/10.1016/j.bjoms.2022.10.008

Stefano, D.C., Francesca, D.A., Matteo, A., Jamshir, S., Michele, F., Brauner, E., 2019. Prosthetic Rehabilitation with Use of Palatal Augmentation Prosthesis in Patients Affected by Functional Limitations of the Tongue. Journal of International Dental and Medical Research 12, 607–611.

Stolte, K., Danker, K., Witt, M., Ebhardt, H., Dommisch, H., 2024. Upregulation of psoriasin/S100A7 correlates with clinical severity in patients with oral lichen planus. CLINICAL ORAL INVESTIGATIONS 28. https://doi.org/10.1007/s00784-024-05717-z

Stuani, V.T., Santos, P.S.S., Damante, C.A., Zangrando, M.S.R., Greghi, S.L.A., Rezende, M.L.R., Sant’Ana, A.C.P., 2018. Oral health impact profile of head and neck cancer patients after or before oncologic treatment: an observational analytic case-control study. Support Care Cancer 26, 2185–2189. https://doi.org/10.1007/s00520-018-4066-8

Tesic, M., Cankovic, M., Jevtic, M., Stevanovic, D., 2020. Validation of the oral health impact profile - 14 in patients with head and neck cancer. Medicina oral, patologia oral y cirugia bucal 25, e739–e744. https://doi.org/10.4317/medoral.23765

Wang, S., Yin, S., Zhang, Z., Su, X., Xu, Z., 2019. Quality of Life After Oral Cancer Resection and Free Flap Reconstruction. Journal of Oral and Maxillofacial Surgery 77, 1724–1732. https://doi.org/10.1016/j.joms.2019.02.029

Warhekar, S., Pimpale, G., Warhekar, A., Ingole, R., Ingole, Y., 2024. Assessing the Impact of Precancerous Lesions on Oral Health-Related Quality of Life: A Study at Tertiary Care Hospital. JOURNAL OF PHARMACY AND BIOALLIED SCIENCES 16, S165–S167. https://doi.org/10.4103/jpbs.jpbs_439_23

Westgaard, K.L., Hynne, H., Amdal, C.D., Young, A., Singh, P.B., Chen, X., Rykke, M., Hove, L.H., Aqrawi, L.A., Utheim, T.P., Herlofson, B.B., Jensen, J.L., 2021. Oral and ocular late effects in head and neck cancer patients treated with radiotherapy. Sci Rep 11, 4026. https://doi.org/10.1038/s41598-021-83635-w

Winter, A., Rasche, E., Hartmann, S., Schmitter, M., Kübler, A., Manuel, K., Schulz, S.M., 2021. Validation of the German-language version of the Liverpool Oral Rehabilitation Questionnaire version 3 and evaluation of oral-health-related quality of life among patients with squamous cell carcinoma of the head and neck. Journal of Cranio-Maxillofacial Surgery 49, 1081–1087. https://doi.org/10.1016/j.jcms.2021.06.007

Winter, A., Schulz, S.M., Schmitter, M., Müller-Richter, U., Kübler, A., Kasper, S., Hartmann, S., 2023. Comprehensive Geriatric Assessment and Quality of Life Aspects in Patients with Recurrent/Metastatic Head and Neck Squamous Cell Carcinoma (HNSCC). JCM 12, 5738. https://doi.org/10.3390/jcm12175738

Witsell, D.L., Stinnett, S., Chambers, M.S., 2012. Effectiveness of cevimeline to improve oral health in patients with postradiation xerostomia. Head & Neck 34, 1136–1142. https://doi.org/10.1002/hed.21894

Wolff, T J, Leeper, H.A, Gratton, D. G, Doyle, P.C, 2004. The Psychosocial Aspects of Prosthetic Use Scale (PAPUS): Preliminary Data. Journal of Speech-Language Pathology and Audiology 28.

Xiao, Y., Liang, Y., Yang, L., Yang, W., Liao, G., 2019. Long-Term Quality of Life in Patients With Maxillofacial Malignancies Who Have Undergone Craniofacial Resection: A Cross-Sectional Survivorship Study. Journal of Oral and Maxillofacial Surgery 77, 2573–2583. https://doi.org/10.1016/j.joms.2019.05.025

Xu, Q., Wang, S.-M., Liu, Y.-H., Yin, S.-C., Su, X.-Z., Xu, Z.-F., 2022. Comparison between the radial forearm and groin soft tissue free flaps for reconstruction in patients with oral cavity cancer: a quality of life analysis. International Journal of Oral and Maxillofacial Surgery 51, 1289–1295. https://doi.org/10.1016/j.ijom.2022.04.011

Yanamoto, S., Soutome, S., Murata, M., Kawakita, A., Yamaguchi, E., Yoshida, K., Kurogi, T., Kuroshima, S., Murata, H., Sawase, T., Umeda, M., 2020. Efficacy of silicone soft reliner on the obturator prosthesis after maxillectomy for oral malignant tumors: A single‐arm prospective interventional study. Clinical & Exp Dental Res 6, 612–617. https://doi.org/10.1002/cre2.326

Yang, J, Yang, L., Han, Q., Zhang, Y., Tao, Z., Zhou, Y., Zhang, P., Wang, R., Sun, B., He, J., Gao, J., 2023. The dose limits of teeth protection for patients with nasopharyngeal carcinoma undergoing radiotherapy based on the early oral health-related quality of life. OPEN MEDICINE 18. https://doi.org/10.1515/med-2023-0673

Yang, Jing, Yang, L., Han, Q., Zhang, Y., Tao, Z., Zhou, Y., Zhang, P., Wang, R., Sun, B., He, J., Gao, J., 2023. The dose limits of teeth protection for patients with nasopharyngeal carcinoma undergoing radiotherapy based on the early oral health-related quality of life. Open Medicine 18, 20230673. https://doi.org/10.1515/med-2023-0673

Yang, W., Zhao, S., Liu, F., Sun, M., 2014. Health-related quality of life after mandibular resection for oral cancer: Reconstruction with free fibula flap. Medicina Oral, Patologia Oral y Cirugia Bucal 19, 414–418. https://doi.org/10.4317/medoral.19399

Yuan, Y., Zhang, P., He, W., Li, W., 2016. Comparison of Oral Function: Free Anterolateral Thigh Perforator Flaps Versus Vascularized Free Forearm Flap for Reconstruction in Patients Undergoing Glossectomy. Journal of Oral and Maxillofacial Surgery 74, 1500.e1-1500.e6. https://doi.org/10.1016/j.joms.2016.03.039

Yusa, K., Yamanouchi, H., Yoshida, Y., Ishikawa, S., Sakurai, H., Iino, M., 2017. Evaluation of quality of life and masticatory function in patients treated with mandibular reconstruction followed by occlusal rehabilitation with dental implants: A preliminary report. Journal of Oral and Maxillofacial Surgery, Medicine, and Pathology 29, 499–503. https://doi.org/10.1016/j.ajoms.2017.06.004

Zeman-Kuhnert, K., Gaggl, A., Bottini, G., Wittig, J., Zimmermann, G., Steiner, C., Lauth, W., Brandtner, C., 2024. Long-Term Outcomes of Dental Rehabilitation and Quality of Life after Microvascular Alveolar Ridge Reconstruction in Patients with Head and Neck Cancer. JOURNAL OF CLINICAL MEDICINE 13. https://doi.org/10.3390/jcm13113110

Zhang, S., Wu, S., Liu, L., Zhu, D., Zhu, Q., Li, W., 2020. Assessment of Quality of Life of Free Anterolateral Thigh Flap for Reconstruction of Tissue Defects of Total or Near-Total Glossectomy. Journal of Oncology 2020, 1–5. https://doi.org/10.1155/2020/2920418

Zhu, L., Zhang, J., Chen, W., Svensson, P., Wang, K., 2021. Sensory recovery and oral health-related quality of life following tongue reconstruction using non-innervated radial forearm free flaps. Oral Oncology 121, 105471. https://doi.org/10.1016/j.oraloncology.2021.105471

Zhu, L., Zhang, J., Song, X., Hou, W., Wu, S., Chen, W., Svensson, P., Wang, K., 2017. Sensory recovery of non‐innervated free flaps and nasolabial island flaps used for tongue reconstruction of oncological defects. J of Oral Rehabilitation 44, 736–748. https://doi.org/10.1111/joor.12510

Zucoloto, M.L., Shibakura, M.E.W., Pavanin, J.V., Garcia, F.T., Da Silva Santos, P.S., Maciel, A.P., De Barros Gallo, C., Souza, N.V., Innocentini, L.M.A.R., Humberto, J.S.M., Motta, A.C.F., 2019. Severity of oral lichen planus and oral lichenoid lesions is associated with anxiety. Clin Oral Invest 23, 4441–4448. https://doi.org/10.1007/s00784-019-02892-2
